# Chromosome segregation during spermatogenesis occurs through a unique center-kinetic mechanism in holocentric moth species

**DOI:** 10.1371/journal.pgen.1011329

**Published:** 2024-06-24

**Authors:** Clio Hockens, Hernan Lorenzi, Tricia T. Wang, Elissa P. Lei, Leah F. Rosin

**Affiliations:** 1 Unit on Chromosome Dynamics, Division of Developmental Biology, *Eunice Kennedy Shriver* National Institute of Child Health and Human Development, National Institutes of Health, Bethesda, Maryland, United States of America; 2 TriLab Bioinformatics Group, National Institute of Diabetes and Digestive and Kidney Diseases, National Institutes of Health, Bethesda, Maryland, United States of America; 3 Nuclear Organization and Gene Expression Section; Laboratory of Biochemistry and Genetics, National Institute of Diabetes and Digestive and Kidney Diseases, National Institutes of Health, Bethesda, Maryland, United States of America; Stowers Institute for Medical Research, UNITED STATES

## Abstract

Precise regulation of chromosome dynamics in the germline is essential for reproductive success across species. Yet, the mechanisms underlying meiotic chromosomal events such as homolog pairing and chromosome segregation are not fully understood in many species. Here, we employ Oligopaint DNA FISH to investigate mechanisms of meiotic homolog pairing and chromosome segregation in the holocentric pantry moth, *Plodia interpunctella*, and compare our findings to new and previous studies in the silkworm moth, *Bombyx mori*, which diverged from *P*. *interpunctella* over 100 million years ago. We find that pairing in both *Bombyx* and *Plodia* spermatogenesis is initiated at gene-rich chromosome ends. Additionally, both species form rod shaped cruciform-like bivalents at metaphase I. However, unlike the telomere-oriented chromosome segregation mechanism observed in *Bombyx*, *Plodia* can orient bivalents in multiple different ways at metaphase I. Surprisingly, in both species we find that kinetochores consistently assemble at non-telomeric loci toward the center of chromosomes regardless of where chromosome centers are located in the bivalent. Additionally, sister kinetochores do not seem to be paired in these species. Instead, four distinct kinetochores are easily observed at metaphase I. Despite this, we find clear end-on microtubule attachments and not lateral microtubule attachments co-orienting these separated kinetochores. These findings challenge the classical view of segregation where paired, poleward-facing kinetochores are required for accurate homolog separation in meiosis I. Our studies here highlight the importance of exploring fundamental processes in non-model systems, as employing novel organisms can lead to the discovery of novel biology.

## Author summary

In order for genetic information to be accurately passed on from one generation to the next, maternal and paternal chromosome copies (homologs) must be separated from each other when making egg and sperm cells in a process called meiosis. To facilitate this equal division, homologous partners pair up from end to end, undergo an exchange of genetic information, and then large protein complexes called kinetochores are formed to enable chromosome segregation. Here, we investigate how homologs pair and how kinetochores form during the formation of sperm in two different moth species: *Bombyx mori* (the silkworm moth) and *Plodia interpunctella* (the pantry moth). Using a combination of microscopy approaches, we observe that homologs seem to pair via interactions at chromosome ends. Conversely, we found that kinetochores form at chromosome centers, away from chromosome ends. This is true even when chromosomes are folded into different configurations. These central kinetochores are functional and able to recruit microtubules, the structures that physically pull chromosomes apart for segregation. How chromosome centers are specified to be kinetochore-recruitment sites in meiosis, and what causes the formation of different chromosome folding configurations, are areas of future study.

## Introduction

Chromosome segregation is an essential part of cell division in both somatic and germline tissues. In the specialized meiotic cell divisions in the germline, chromosomes undergo an intricate multi-step alignment process before chromosome segregation can occur. First, chromosomes become more linear, then homologous chromosomes (maternal and paternal copies of the same chromosome) find each other and pair from end-to-end. This end-to-end pairing allows for crossover recombination and the formation of chiasmata, which link homologous chromosomes together. These linkages assist in the proper bi-orientation of homologs on the metaphase I spindle, where homologous kinetochores must face opposite spindle poles for accurate chromosome segregation. Each of these steps needs to be executed accurately to avoid chromosome segregation defects during meiosis such as nondisjunction.

Even though the processes that occur during early meiosis are largely conserved (homolog pairing, recombination, and segregation), researchers have found that different molecular mechanisms mediate these processes in different species. For example, in both male and female meiosis in the fruit fly *Drosophila melanogaster*, centromeres and pericentric heterochromatin have been implicated in early meiotic pairing events at (or even before) meiotic entry [1–8], and centromeres also mediate chromosome segregation at metaphase I (reviewed in [6,9]). In *Caenorhabditis elegans*, chromosome-specific sequences and their *cis*-acting binding partners form “pairing centers” at the distal regions of chromosomes (arm-like regions) that facilitate homolog recognition [10–14], and chromosome segregation is mediated through a mechanism where telomeres face the spindle poles and kinetochore cups form around the chromosomes (reviewed in [15,16]). Despite these findings, how homologs find each other in three-dimensional nuclear space and distinguish between homologous partners and heterologous chromosomes is unclear in most other species. Additionally, the mechanisms mediating chromosome bi-orientation and segregation during meiosis I are still not well understood, specifically in holocentric species (where kinetochores form all along the length of the chromosome during mitosis).

Holocentric species, like moths and *C*. *elegans*, face distinct challenges during meiosis compared to mitosis [17,18]. Since recombination is typically suppressed in centromere-proximal regions of chromosomes across species [19–21], it is reasonable to predict that maintaining the holocentric structure during meiosis could largely inhibit recombination. Additionally, centromeres must be bi-orientated on the metaphase plate during both meiosis I and meiosis II for the proper segregation of chromosomes [22,23]. If recombination does occur and results in a cruciform-shaped bivalent (a cross-shaped structure with long and short arms perpendicular to each other), having centromeres all along the length of the chromosome would mean centromeres face many different directions, which could lead to failed bi-orientation [17]. Therefore, holocentric species must evolve mechanisms of chromosome segregation during meiosis that are distinct from those used during mitosis. Three meiosis-specific mechanisms have been identified to date in holocentric species: 1) inverted meiosis, where sister chromatids are segregated before homologs and the holocentric structure is at least partially maintained [24–27], 2) telokinetic (or telokinetic-like) meiosis, where telomeres or distal chromosome regions become kinetochore-recruitment sites [28,29], and 3) chromosome restructuring as seen in *C*. *elegans*, where telomeres face the spindle pole and kinetochore cups form around chromosomes [16,29–34].

Our previous work in the silkworm moth, *Bombyx mori*, suggested that gene-rich chromosome domains near telomeres facilitate homolog recognition and that telomeres face the spindle poles for chromosome segregation in the male germline [35]. However, whether these findings apply to all Lepidopteran insects (of which there are almost 200,000 spanning more than 120 million years of evolution) is unknown. Here, we employ a combination of Oligopaint DNA FISH and immunofluorescence to investigate meiotic chromosome dynamics in the pantry moth *Plodia interpunctella* and compare our findings to new and previous work in *Bombyx*. *Plodia* is a promising new laboratory model for genetics and genomics in Lepidoptera. While *Bombyx* have been used for decades as a model Lepidopteran system, creating transgenic *Bombyx* is extremely challenging and the feeding and space demands of rearing these large insects make them sub-optimal for laboratory research. *Plodia*, on the other hand, are small, their embryos are easy to inject, and several recent studies have demonstrated high success in creating transgenic strains using CRISPR and *PiggyBac* technologies [36,37].

Our studies here demonstrate the suitably of using *Plodia* as a model system for studying both conserved and divergent aspects of meiotic chromosome biology. Using Oligopaints to label multiple domains along individual chromosomes in *Plodia* larval testes, we find that pairing in *Plodia* spermatogenesis is initiated at gene-rich chromosome ends, similar to *Bombyx*. Additionally, both *Bombyx* and *Plodia* form rod shaped cruciform-like bivalents at metaphase I. However, while *Bombyx* bivalents always have telomeres oriented toward spindle poles at metaphase I in spermatogenesis, we find that *Plodia* bivalents can be either “telomere-oriented” (telomeres facing spindle poles) or “center-oriented” (chromosome centers facing spindle poles). Intriguingly, we find that both *Plodia* and *Bombyx* form kinetochores at non-telomeric loci towards chromosome centers, even when telomeres are oriented toward spindle poles. We term this “center-kinetic” chromosome segregation. These central kinetochores do not seem to pair between sister chromatids, resulting in metaphase I structures with four distinct kinetochore foci. Despite the spacing between kinetochores, we observe clear co-orientation mediated by end-on microtubule attachments. This mode of chromosome segregation appears to be a completely distinct mechanism from that employed by these moths during mitosis, where they are holocentric, and it is distinct from other previously described mechanisms of meiotic segregation in holocentric species. Together, our studies reveal that unique mechanisms dictate chromosome segregation dynamics in Lepidopteran spermatogenesis.

## Results

### Visualizing chromosomes in the male germline of *Plodia interpunctella* using Oligopaints

**Fig 1:**
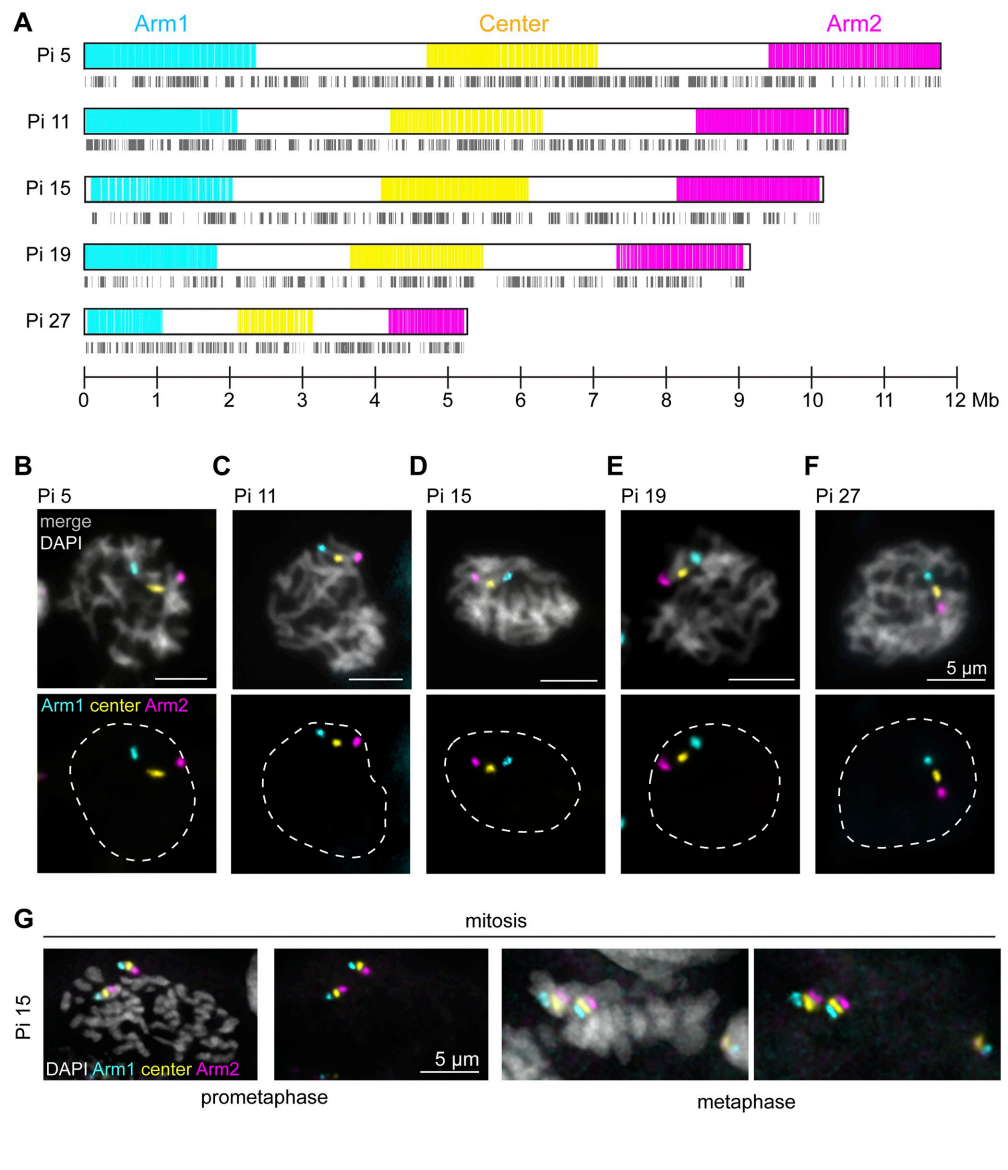
Oligopaint design and specificity in *Plodia interpunctella*. A) Schematic of Oligopaint design for *Plodia* ch5, 11, 15, 19, and 27. Gray lines below indicate the position of genes. B-F) Pachytene nucleus from *Plodia* 5^th^ instar larval testis labeled with Oligopaints for *Plodia* ch5 (B), 11 (C), 15 (D), 19 (E), or 27 (F). Arm1 probe is shown in cyan. Center probe is shown in yellow. Arm2 probe is shown in magenta. DAPI is shown in gray. Scale bar = 5 μm. Dotted line indicates the approximate nuclear edge. G) Mitotic prometaphase (left) and mitotic metaphase (right) cells from *Plodia* 5^th^ instar larval testis squash labeled with Arm1, Center, and Arm2 probes for *Plodia* ch15 as in B.

To visualize *Plodia* chromosomes during meiosis, we designed and generated Oligopaint libraries targeting five of the 30 *Plodia* autosomes. Since our previous studies in *Bombyx* suggested chromosome size and gene distribution might influence meiotic chromosome dynamics, we designed probes targeting three large, one medium, and one small *Plodia* chromosome, with varying distributions of genes across the chromosomes (ch5, 11, 15, 19, and 27; Fig 1A). Our paint design here focused on creating a three-stripe pattern across these chromosomes, with probes labeling the single-copy sequences in both distal arm-like regions (designated Arm1 or Arm2 probes–hereafter referred to as “arms”) and the middle of the central region (Center probe, Fig 1A). This allowed us to not only visualize single chromosomes as they progress through meiosis but also to analyze the dynamics of specific sub-chromosomal domains as previously described [35].

The genome we originally used to design the ch5, 19, and 27 paints was in a draft state (assembled into large, chromosome size scaffolds with many unmapped contigs) [38]. Since then, a new chromosome-level genome assembly has been released, which was used to design the paints for ch11 and 15 [39]. Mapping these two genome versions to each other revealed high conservation between the draft genome and chromosome-level assembly overall, with especially high fidelity for our five targeted chromosomes (S1A–S1F Fig). Additionally, mapping of the new *Plodia* genome to the latest release of the *B*. *mori* genome revealed a high level of synteny between these two species (S1G Fig). As further verification of the accuracy of the *Plodia* genome assemblies, stripe Oligopaints applied to pachytene nuclei from 5^th^ instar larval testes show the expected linear pattern from one telomere to the other of each chromosome (Fig 1B–1F), as do mitotic cells from the germline (Fig 1G). Together, these assays validate the *P*. *interpunctella* genome assemblies and demonstrate the specificity of our Oligopaints.

### Homolog pairing is initiated at gene-rich chromosome ends in *Plodia*

**Fig 2:**
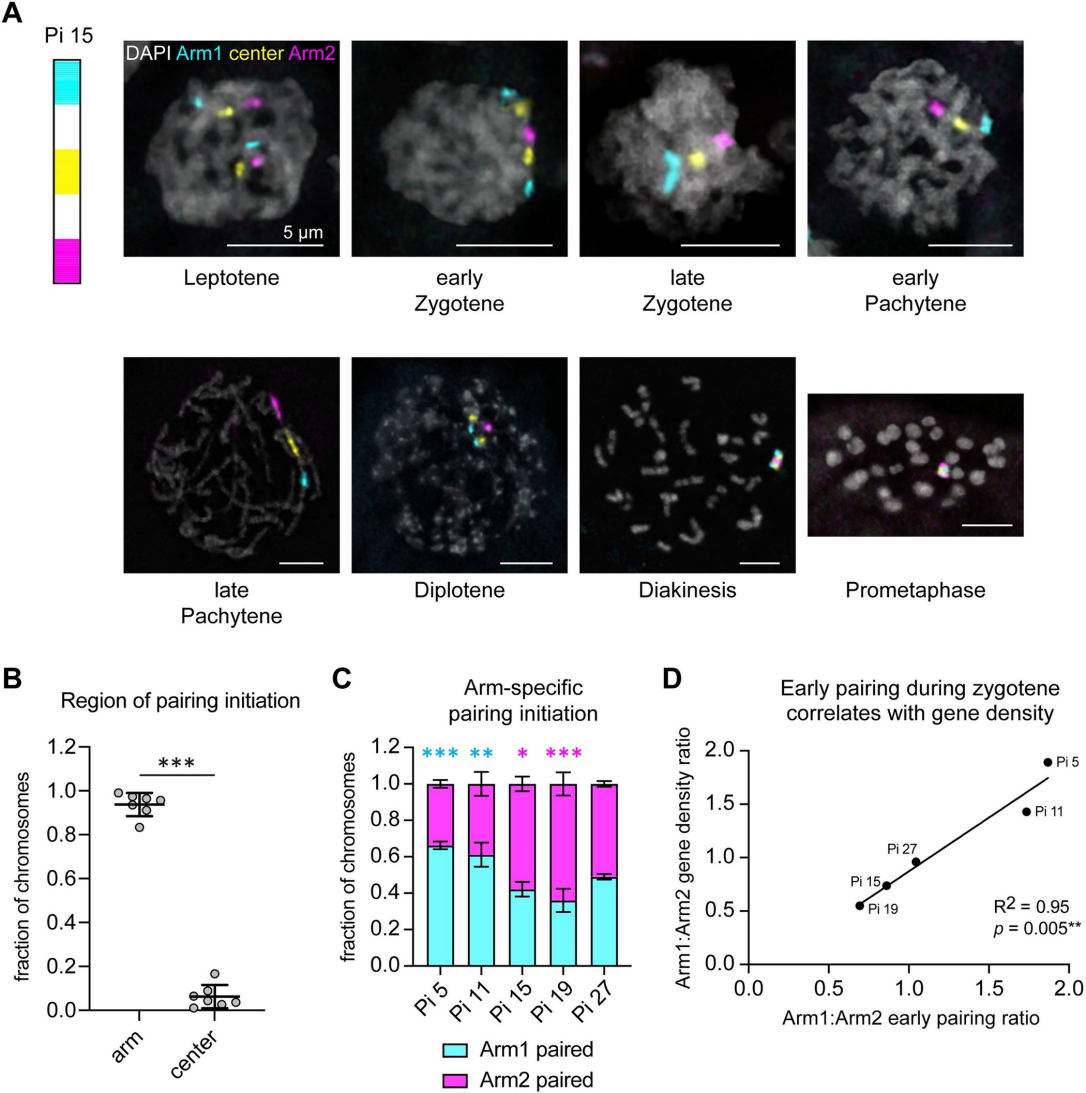
Meiotic pairing is initiated at gene-rich chromosome ends in *Plodia*. A) Left: Schematic of Plodia ch15 Oligopaints. Right: Nuclei labeled with *Plodia* ch15 stripe paints representing the various chromosome configurations observed in Prophase I (Leptotene, Zygotene, Pachytene, Diplotene, Diakinesis) and Prometaphase I from *Plodia* 5^th^ instar larval testes squashes. Arm1 probe is shown in cyan. Center probe is shown in yellow. Arm2 probe is shown in magenta. DAPI is shown in gray. Scale bar = 5 μm. B) Beeswarm plot showing the fraction of chromosomes with the designated chromosome regions (X-axis) initiating pairing. Each dot represents the average data for a single chromosome. Mid-line = mean. Error bars = SD. *** *p* < 0.0001. Fisher’s exact test. For B-D, data for each chromosome are from an average of four testes from across two-three replicate experiments (two testes each for two distinct FISH experiments). C) Bar graph showing mean and standard deviation of arm-specific chromosome pairing initiation for *Plodia* ch5, 11, 15, 19, and 27. *** *p* < 0.0001, ** *p* = 0.0005, * *p* = 0.01. Fisher’s exact test. For B and C, pairing was determined by the number of FISH signals present, with a single (continuous) FISH signal indicating “paired” and two distinct FISH signals detectable by eye indicating “unpaired.” This was quantified in very early zygotene cells, where only one of the three FISH signals was paired and the other two remained unpaired. D) Scatter plot showing Arm1:Arm2 pairing initiation ratio (X-axis) versus Arm1:Arm2 gene density ratio (Y-axis). Gene density for each probe domain is calculated as the base pairs covered by genes out of total base pairs painted by the Oligopaint. Linear regression used to calculate the line of best fit, R^2^, and *p*-value. For B-D, n > 300 cells across four replicates for Pi 5, 19, and 27. n > 200 cells across three replicates for pi 11 and 15.

Our previous studies in *Bombyx* revealed that meiotic homolog pairing in the male germline is initiated at gene-rich chromosome ends. To test if this mechanism is conserved in *Plodia*, which diverged from *Bombyx* over 100 million years ago, we labeled one chromosome at a time using our stripe Oligopaints described above to quantify which chromosome region (arms or center) pairs first as cells progress through early meiotic prophase (Fig 2A) in early 5^th^ instar larval testes. Analysis of pairing between Arm1, Arm2, and Center domain probes for all painted chromosomes revealed that like *Bombyx*, pairing in *Plodia* is initiated at distal chromosome arm domains more frequently than the central domain (Fig 2B).

Interestingly, when we broke these pairing data down further to examine whether pairing is preferentially initiated at one arm compared to the other on each chromosome, we found that both arms can initiate pairing but there is sometimes a bias towards one arm (Fig 2C). For example, Arm1 on ch5 is significantly more likely to initiate pairing than Arm2. Conversely, Arm2 on ch19 is significantly more likely to initiate pairing compared to Arm1 (Fig 2C). In *Bombyx*, we previously observed a similar bias in pairing initiation between chromosome arms and found that pairing initiation is strongly correlated with gene density, with gene-rich arms pairing earlier than gene-poor arms [35]. This same correlation was observed in our data from *Plodia* (Figs 2D and S2A), suggesting that gene-rich chromosome arms may initiate pairing broadly in Lepidoptera. Together, we find that pairing initiation occurs at the distal regions of chromosome arms in *Plodia*, with gene-rich arms being more likely to pair before gene-poor arms.

### Bivalent orientation at metaphase I varies from cell to cell in *Plodia*

**Fig 3:**
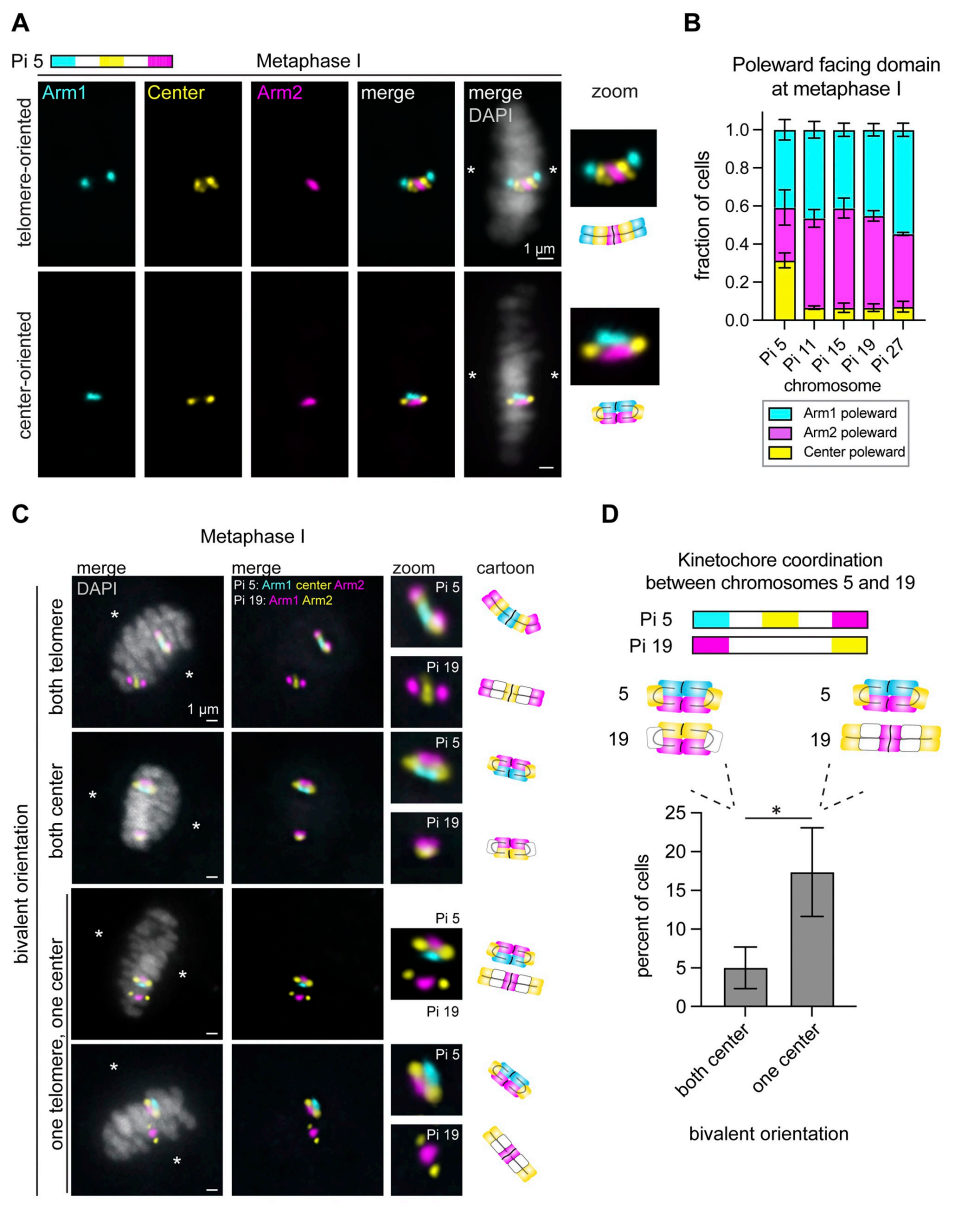
Bivalent orientation is variable from cell to cell at metaphase I in *Plodia* spermatogenesis. A) Representative metaphase I cells from *Plodia* 5^th^ instar larval testes squashes labeled with ch5 Oligopaints. Schematic for Oligopaints is shown above. Arm1 probe is shown in cyan, Center probe in yellow, Arm2 probe in magenta, and DAPI in gray. Scale bar = 1 μm. Asterisks indicate the spindle poles. Zoom and cartoon schematic (bottom) of bivalent orientations are shown on the right. B) Bar graph showing quantification of metaphase I orientation for ch5, 11, 15, 19, and 27. Ch5, n = 233 (30.5% Arm1 facing poleward, 39% Arm2 poleward, 30.5% Center poleward). Ch11, n = 215 (47% Arm1, 48% Arm2, 6% Center). Ch15, n = 308 (42% Arm1, 53% Arm2, 6% Center). Ch19, n = 269 (44% Arm1, 51% Arm2, 5% Center). Ch27, n = 184 (53% Arm1, 40% Arm2, 7% Center). For all, p > 0.05 when comparing Arm1 to Arm2 poleward percentages using Fisher’s exact test. Each FISH assay was performed on n = 3–5 testes each from separate larvae across two distinct FISH experiments. C) Representative metaphase I cells from *Plodia* 5^th^ instar larval testes squashes co-labeled with ch5 Oligopaints and ch19 Oligopaints. Schematic for Oligopaints is shown in C. Scale bar = 1 μm. Asterisks indicate the spindle poles. Right: zoom of individual bivalents and cartoon of bivalent configurations. D) Top: Schematic for Oligopaints used to analyze bivalent coordination between ch5 and ch19 in C. Middle: Cartoon showing both chromosomes in a center-oriented position (left) versus ch5 in a center-oriented configuration and ch19 in telomere-oriented configuration (right). Bottom: bar graph showing mean and standard error of the mean of the percentage of cells showing bivalent coordination between ch5 and 19, as shown in C. Bivalent coordination is defined as both chromosomes showing the same orientation at metaphase I (telomere-oriented/center-oriented). The remaining cells showed both chromosomes in a telomere-oriented configuration. Two testes from two larvae were quantified (two technical and two biological replicates; 4 replicates total), n > 50 cells from each gonad. * *p* = 0.03, Two-tailed Welch’s T-test.

The symmetrical segregation of homologs during meiosis requires specific changes to chromosome structure compared to mitosis to allow for homologs to be properly bi-oriented at metaphase I. Our previous work in *Bombyx* revealed that bivalents at metaphase I are bi-oriented such that homologs remain linked at one distal arm domain while the other arm faces the spindle poles [35]. Here we refer to this bivalent configuration as “telomere-oriented” since the chromosome domain most proximal to the spindle poles is the telomere. To see whether bivalents are also telomere-oriented in spermatogenesis in *Plodia*, we used stripe Oligopaints to visualize the position of chromosome arms and centers at metaphase I. Surprisingly, our analyses revealed that our five analyzed chromosomal bivalents are in a telomere-oriented configuration in only 70–90% of cells (Figs 3A, 3B and S2B). In the remaining 10–30% of cells, the center region of the chromosome faces the spindle pole (center-oriented bivalent; Figs 3A, 3B and S2B). In this center-oriented configuration, the homologs seemingly remain linked by both chromosome arms at metaphase I. Bivalents with either one telomere linked or both telomeres linked can also be observed prior to metaphase I in diakinesis (S3A Fig).

Among the 70–90% of chromosomes in a telomere-oriented configuration at metaphase I, no significant biases were observed for which of the two telomeres face the spindle pole (Fig 3B). For ch5, 40% of chromosomes were in an Arm1-oriented position (Arm1 probe facing the poles) and 28% in an Arm2-oriented position (*p* = 0.3; Fisher’s Exact Test comparing observed to expected, where expected is 50:50). For ch11, 46% were Arm1-oriented and 47% were Arm2 oriented (*p* = 1.0). For ch15, 58% were Arm1-oriented and 36% were Arm2-oriented (*p* = 0.11). For ch19, 45% were Arm1-oriented and 48% were Arm2-oriented (*p* = 0.66). Finally, for ch27, 55% were Arm1-oriented and 38% were Arm2-oriented (*p* = 0.25). This suggests that both telomeres are equally likely to face the spindle poles and orient bivalents at metaphase I.

Interestingly, we do not find strong evidence suggesting that all chromosomes in a cell employ the same bivalent orientation at a given time (not all center-oriented or all telomere-oriented). As the most common bivalent orientation is telomere-oriented, when ch5 and 19 were co-analyzed in single cells, both chromosomes were most often telomere-oriented across a population of cells. Similarly, in a single cell, if one chromosome was found to be center-oriented, the other chromosome was still most often telomere-oriented rather than both being center-oriented (Fig 3C and 3D). Thus, whole cells do not seem to be employing center-orientation across all bivalents at the same time. These data are further supported by FISH with the insect pentameric telomere repeat [40] in diakinesis cells, which show circular bivalents with both arms linked and linear bivalents with one arm linked in the same cells (S3B Fig). To summarize, we find that metaphase I bivalent orientation can vary not only between cells, but between different chromosomes in the same cell, with different chromosome regions facing the spindle pole throughout the cell population.

### Homologs are segregated using a novel “center-kinetic” mechanism at metaphase I in *Plodia*

**Fig 4:**
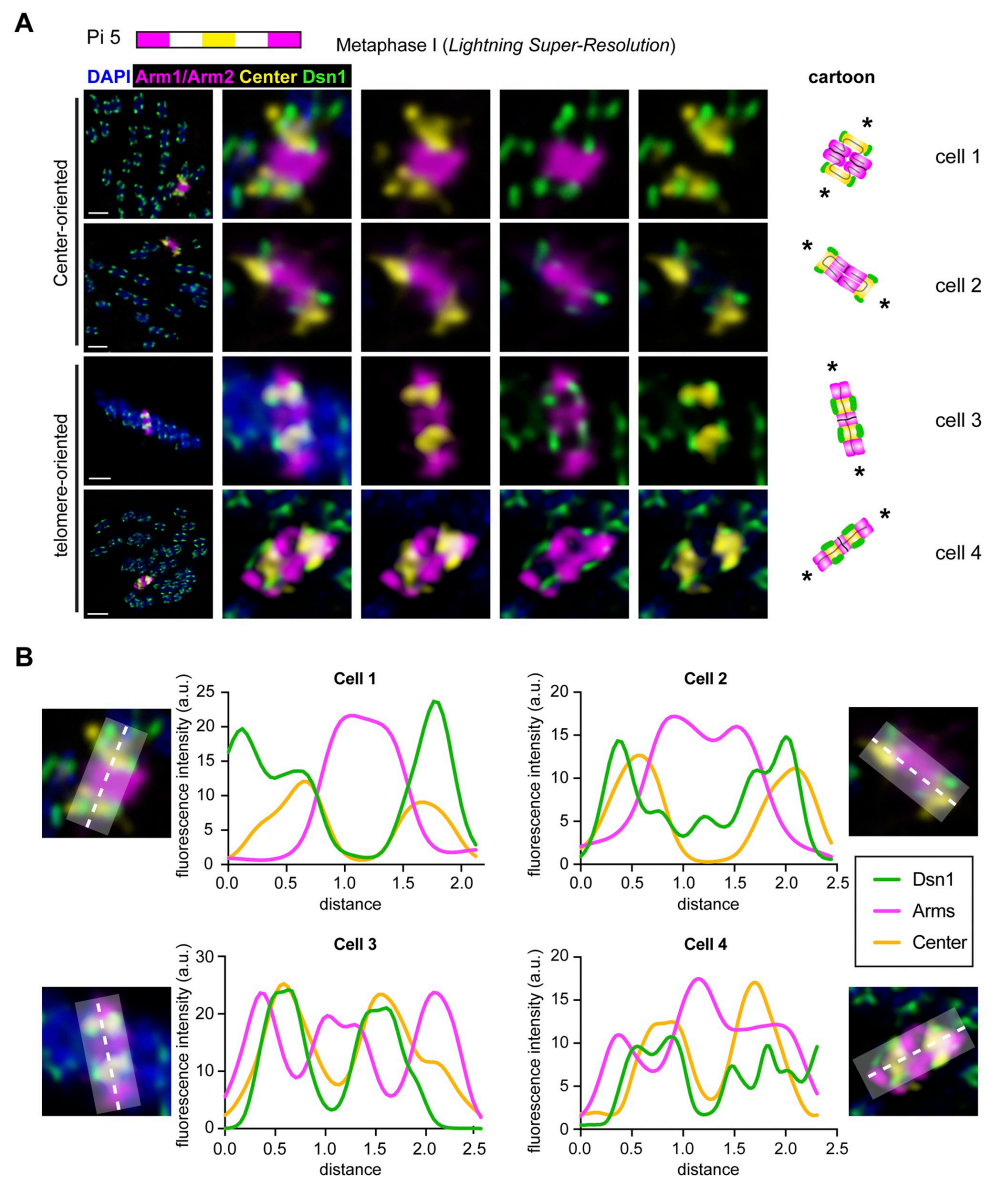
Homolog are segregated through a “center-kinetic” mechanism in *Plodia* spermatogenesis. A) Representative metaphase I cells from *Plodia* 5^th^ instar larval testes squashes labeled with ch5 Oligopaints and Dsn1 IF showing center-oriented chromosomes (top two rows) or telomere-oriented chromosomes (bottom two rows). Schematic for Oligopaints is shown above. Arm1 and Arm2 probes are shown in magenta, Center probe in yellow, Dsn1 IF is shown in green, and DAPI in blue. Scale bar = 2 μm. Columns 2–5 are zooms of painted chromosome in column 1. Cartoon schematic of bivalents are shown on the right. Asterisks indicate the spindle poles. Other Dsn1 signal shown in zoomed panels are kinetochores on other chromosomes. B) Line plots showing signal localization across bivalents of painted chromosomes from A.

The fact that some bivalents are telomere-oriented at metaphase I and some are center-oriented suggested to us that kinetochore position on chromosomes could vary from cell to cell, with kinetochores forming at whichever chromosome region faces the spindle poles. Alternatively, kinetochores could always form along the entire length of the chromosome, maintaining the holocentric structure and ensuring that any chromosome region that faces the poles can direct chromosome segregation. To differentiate between these two possibilities, we performed co-IF-FISH labeling the outer kinetochore protein Dsn1 (a component of the Mis12 complex [41,42]) and ch5 stripes. Both arms of ch5 were labeled in the same color and the center of ch5 in a separate color to distinguish between telomere-oriented and center-oriented bivalents (Figs 4A, S4A and S4B).

Strikingly, instead of either of our predicted models, this assay revealed that kinetochores are restricted to chromosome centers regardless of bivalent orientation (Fig 4A). Specifically, we found that Dsn1 localizes within or at the edges of the center probe of ch5 in all examined metaphase I cells (n = 99 cells across 3 biological replicates). This was true whether ch5 was in a telomere-oriented configuration (n = 72) or a center-oriented configuration (n = 27; Fig 4A). Similar results were seen when examining ch11 (S4C Fig). We term this “center-kinetic segregation”, where kinetochores localize only to the center of the chromosome (not along the distal chromosome arms). To confirm that *Plodia* are holocentric during mitosis and do not always harbor centrally localized kinetochores, we performed mitotic chromosome spreads using PiD2 *Plodia* cultured cells [43] and labeled these with Dsn1 IF (S4D Fig). This experiment validated that *Plodia* are indeed holocentric during mitosis, and centrally-restricted kinetochores are meiosis-specific (possibly even spermatogenesis-specific).

This surprising result led us to wonder about kinetochore position in *B*. *mori* spermatogenesis. Even though *Bombyx* always display telomere-oriented bivalents and kinetochore proteins are seemingly present near chromosome ends at metaphase I [35], we wondered whether the kinetochores are actually further away from telomeres than previously appreciated in *Bombyx*. To test this, we performed the same co-IF-FISH assay with Dsn1 and *Bombyx* ch15 stripe paints in *Bombyx* 4^th^-5^th^ instar larval testes squashes (S5 Fig). To our surprise, this experiment revealed that like *Plodia*, *Bombyx* telomeres are not utilized for kinetochore formation, despite their poleward-facing position. Instead, we observed Dsn1 localization away from telomeres and closer to the chromosome center, just as in *Plodia* (S5 Fig).

Together, these data suggest that moths employ a novel “center-kinetic” segregation mechanism during spermatogenesis, where kinetochore formation is restricted to the center of the chromosome. This is a unique mechanism that differs from the mitotic holokinetic or holocentric segregation mechanism employed by these species. It is also distinct from the previously described meiotic mechanisms in holocentric species: telokinetic, kinetochore cups, and inverted meiosis. Finally, center-kinetic segregation is distinct from a classical “monocentric” segregation mechanism (as observed in flies and mammals) where kinetochores form at the endogenous centromere locus defined by the histone H3-variant CENP-A in every mitotic and meiotic cell.

### Centrally positioned kinetochores recruit microtubules via end-on attachments

We noted that in both *Plodia* and *Bombyx*, two distinct sister kinetochores are present on each homolog, rather than the single, paired kinetochore observed during meiosis in other species [44]. This could pose an issue for proper bi-orientation of homologs, as the two sister kinetochores need to co-orient (be attached to microtubules from the same spindle pole). Sister kinetochore “splitting” as we observed here (Figs 4, S4 and S5) has previously been shown to increase the probability of incorrect microtubule attachments, such as sister kinetochores attaching to microtubules from opposite spindle poles during metaphase I [45,46]. Thus, we wondered whether these separated kinetochores are actually functional and recruit microtubules, or if they are perhaps vestigial and microtubule attachments occur via an alternative mechanism in these systems.

To determine if these Dsn1 foci are positioned at functional kinetochores, we first asked whether we could see other kinetochore proteins at the same central position on chromosomes, suggesting that the full kinetochore complex is present there. To this end, we performed IF with anti-CENP-T antibody, an inner kinetochore protein, and separately with anti-Spc24/25, an outer kinetochore protein (part of the NDC80 complex) [47]. While we did not observe robust CENP-T signal in early meiotic prophase (leptotene), we found that by pachytene, CENP-T localizes all along the length of the chromosome axis, where it colocalizes with other axis proteins like Cohesin (S6A and S6B Fig). While somewhat surprising, this result agrees with recent data on kinetochore formation in *Bombyx mori* female meiosis, which showed kinetochore proteins initially localizing between homologs as part of the bivalent bridge until late metaphase I, when kinetochores switched to their more canonical poleward-facing position [48]. Similarly, in *Plodia* spermatogenesis, we found that by metaphase I, CENP-T was restricted to the center of chromosomes, in agreement with our Dsn1 staining (S6C and S6D Fig). These findings suggest that non-central CENP-T is removed or repositioned to prepare for “center-kinetic” localization at metaphase I. Importantly, we also observed that Spc-24/25 is present at the central chromosome location at metaphase I in *Bombyx* (where we have a specific antibody for this protein; S6E Fig), suggesting that the outer kinetochore is recruited there and supporting a model where central kinetochores are functional.

**Fig 5:**
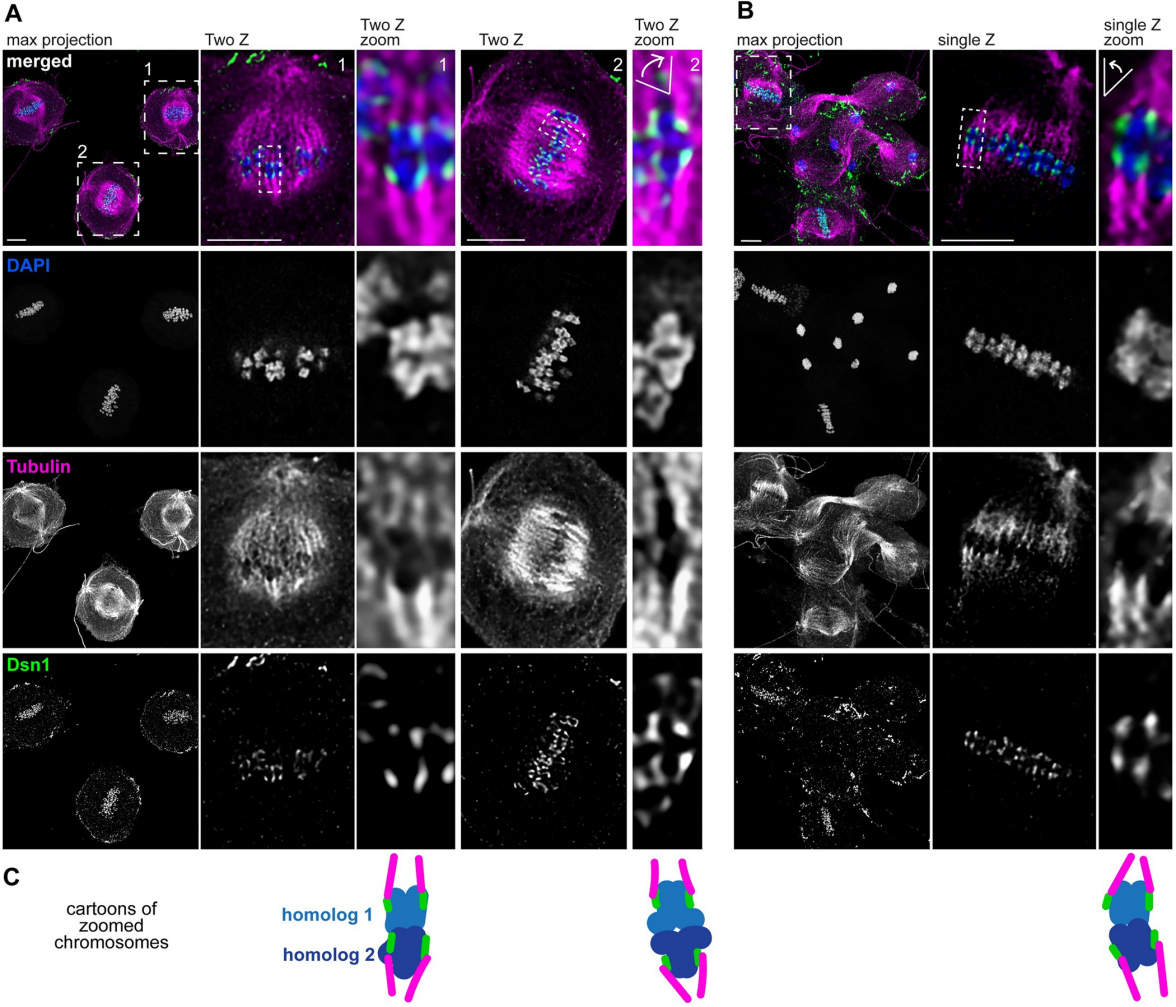
Kinetochores in *Plodia* spermatogenesis recruit microtubules via end-on attachments. A and B) Representative IF images of metaphase I cells from *Plodia* larval testes squashes showing DAPI in blue, microtubules in magenta, and Dsn1 kinetochores in green. Left to Right: a field of cells with entire spindles visible, a single cell zoom, then a single chromosome zoom. Zoomed in cells and chromosomes are indicated by the dashed box in the previous panel. Scale bar = 5 μm. n = 30 metaphase I cells each for two biological replicates (~60 cells total) were examined. 100% showed direct kinetochore-microtubule attachments and not lateral attachments. C) Cartoon schematic of single chromosome zooms shown above in A and B. All images were acquired with a Stellaris confocal and super-resolution processing.

To determine whether these kinetochores forming at the center of chromosomes recruit microtubules, we performed IF with an anti-tubulin cocktail (see methods) and anti-Dsn1 on *Plodia* larval testes squashes to visualize kinetochores and microtubules at metaphase I. This revealed clear end-on attachments of microtubule bundles to kinetochores at metaphase I, even for bivalents where kinetochores were clearly forming away from the most poleward portion of the chromosome (Fig 5). Furthermore, sister kinetochores appeared to be co-oriented in all observed cells, with bivalents seemingly positioned such that homologs (not sisters) face opposite poles (Fig 5). These data mirror our previous findings of microtubule attachments at metaphase I in *Bombyx* spermatocytes [35]. Together, these findings support the notion that the centrally-restricted kinetochores we observe in moth spermatogenesis are functional and represent a novel mode of meiotic chromosome segregation for holocentric species.

## Discussion

Here, we employed the Oligopaint DNA FISH technology to characterize meiotic chromosome dynamics in the holocentric pantry moth *Plodia interpunctella* for the first time. By visualizing single chromosomes at sub-chromosomal resolution, we found that meiotic homolog pairing during spermatogenesis is initiated at gene-rich chromosome ends. This finding mirrors our previous studies in *Bombyx mori*, suggesting that this mechanism may be broadly conserved in Lepidopteran insects. Whether or not transcription, the transcription machinery, or transcripts themselves facilitate homolog recognition and pairing in Lepidoptera is an area for future study. Indeed, transcription has been associated with meiotic pairing in other species, such as *S*. *pombe* [49,50], and with somatic pairing in species from flies to humans [51–54], suggesting that pairing may be regulated by similar mechanisms across species. How gene density may influence other early meiotic events such as the formation of double-strand breaks, and whether breaks are actually required for pairing in *Plodia* spermatogenesis, are areas of ongoing investigation.

Intriguingly, while pairing is initiated at chromosome ends, chromosome ends are not used for chromosome segregation at metaphase I in *Plodia*. Instead, we observed that bivalents can be positioned in either a telomere- or center-oriented configuration at metaphase I (with telomeres or centers facing the spindle poles, respectively). Furthermore, chromosome centers act as kinetochore-recruitment sites in *Plodia* spermatogenesis. A similar mechanism was observed in *Bombyx* spermatogenesis, despite the fact that telomeres are always oriented toward spindle poles in this species. This mechanism of segregation differs from the cup-based segregation mechanism observed in the holocentric nematode *C*. *elegans* [15,16] and the inverted meiosis observed in some holocentric plants [17].

How telomere- and center-oriented bivalents are formed remains unclear. Like *Bombyx*, *C*. *elegans* harbor exclusively telomere-oriented bivalents at metaphase I. In *C*. *elegans*, this structure is achieved via crossover regulation. Crossovers (COs) in *C*. *elegans* are almost always limited to one per chromosome, and these COs form in one of the two distal arm-like regions [55–60]. The arm-like region of the homologs with the chiasma remains connected at metaphase I and is positioned along the metaphase plate, while the opposite arm-like region points out toward the spindle poles [16,31,61–63]. A similar CO-based mechanism could explain the formation of telomere- and center-oriented bivalents in moths, but this has yet to be experimentally tested. For example, the telomere-oriented bivalents observed for all *Bombyx* and some *Plodia* chromosomes could be the result of a single distal CO, consistent with studies suggesting only a single CO occurs per chromosome in Lepidoptera [64–66]. Conversely, our observation of some center-oriented bivalents with both chromosome arms remaining linked at the center of the metaphase I plate in *Plodia* would suggest the formation of two COs between these homologs. Support for the formation of double COs in Lepidoptera comes from recent data showing that some chromosomes, indeed, harbor two COs in *Papilio* butterflies [67].

Another possibility is that homologs in *Plodia* remain linked at metaphase I by non-crossover mechanisms, such as those that have been reported in *Drosophila* males, *Bombyx* females, and *C*. *elegans* [48,68–74]. The fact that we do not observe a strong correlation between chromosome length and center-oriented bivalents would, indeed, support a model where these center-oriented bivalents are not exclusively forming by double crossovers (which we would expect to be more frequent for longer chromosomes).

While it seems most likely to us that the center-oriented bivalents observed at metaphase I represent the homologs being physically linked at both chromosome ends, it is also possible that these structures are instead a transient state. In this scenario, the telomere-oriented configuration would be a precursor to the center-oriented configuration, which is then achieved by the central kinetochore domains being pulled toward the poles via the meiotic spindle, flipping the homologs and resulting in a structure with both arm domains facing each other at the metaphase I plate. However, as we observe both center- and telomere-oriented bivalent structures already in diakinesis (S3A and S3B Fig), and they persist through alignment at metaphase I (Figs 3 and 4), we find this is possibility unlikely. Additionally, early anaphase I cells from *Bombyx* testes still largely show telomere-oriented chromosomes, and it is not until late anaphase that these bivalents “flip” to a center-outward configuration (S7 Fig). These results suggest that the center-oriented bivalents at *Plodia* metaphase I are likely not a transition state (since a transition to center-out does not happen until late anaphase).

**Fig 6:**
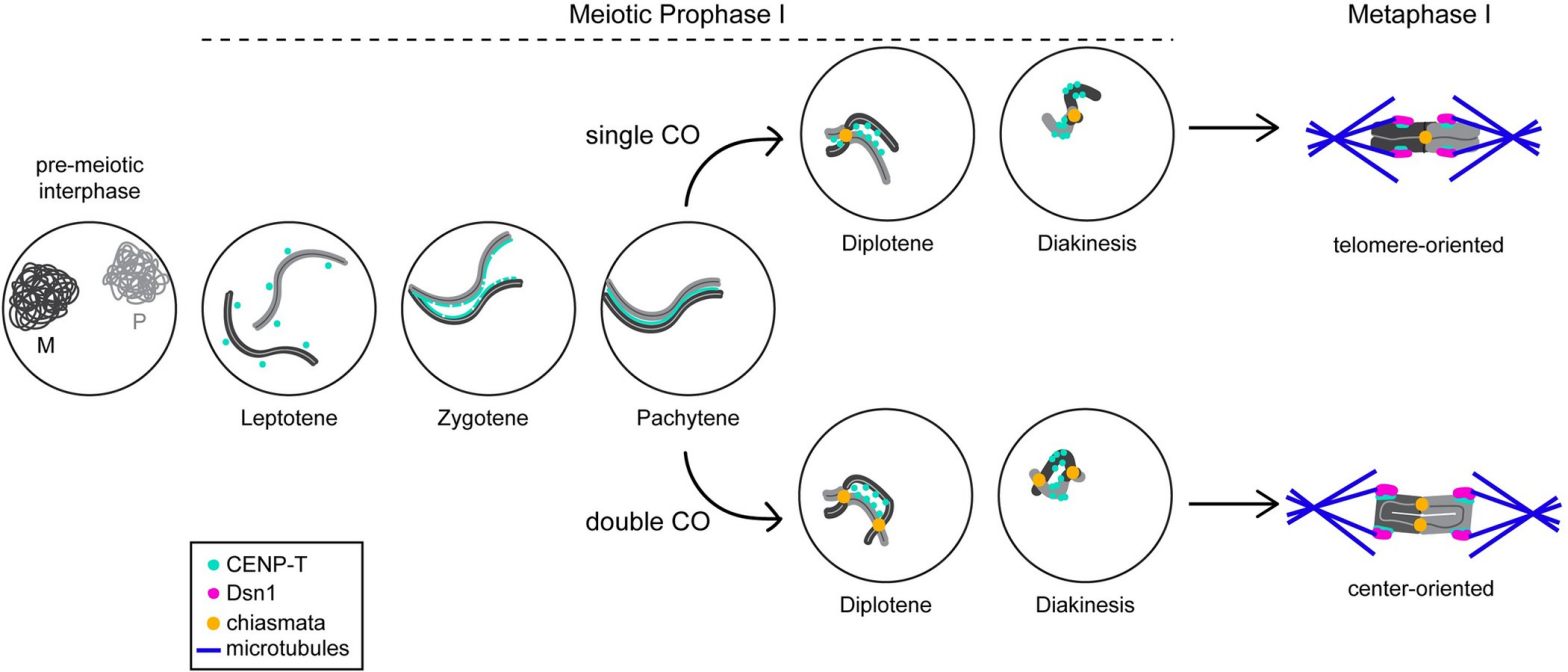
Speculative model for center-kinetic chromosome segregation. Schematic showing one possible model for pairing and chromosome segregation during moth spermatogenesis. Homologs come together at gene-rich regions during zygotene. At the same time, CENP-T is assembled along the chromosome axis, where it remains through pachytene. During pachytene, either single or double crossovers may occur, leading to the formation of center-oriented (bottom) or telomere-oriented (top) bivalents, respectively. Regardless of bivalent orientation, CENP-T and other kinetochore proteins like Dsn1 become restricted to chromosome centers and recruit the meiotic spindle via end-on attachments for chromosome segregation.

Regardless of how homologs are associated at metaphase I, we surprisingly found that kinetochores always form toward the center of chromosomes. How kinetochore position is defined and regulated during spermatogenesis in moths is yet to be thoroughly explored. We propose a highly speculative model (Fig 6) where CENP-T is loaded with synaptonemal complex proteins early in meiotic prophase I. Since CENP-T shows low retention rates at chromosome regions with active gene expression in mitotic cells [75], we propose that active transcription at telomere-proximal regions in meiosis (which may or may not facilitate homolog recognition) could lead to the loss of CENP-T from the distal regions of chromosomes. In this scenario, the maintained CENP-T in the center of chromosomes would go on to define the kinetochore position at metaphase I. In this model, COs would largely occur within the distal arm regions of the chromosome, away from this newly defined “centromere”, which is consistent with the various structures we observed at metaphase I. Such a mechanism would be in agreement with the suppression of COs near centromeres (reviewed in [19]), and with COs being more likely to form in open chromatin regions compared to heterochromatin [76,77]. Single versus double COs could then lead to a telomere-or center-oriented bivalent, respectively, as described above.

Finally, we predict that the unpaired sister kinetochores observed at metaphase I in moths provides some flexibility to the orientation of bivalents. Studies have shown that in Lepidopteran hybrids, inverted meiosis (separation of sisters before homologs) tends to be favored, presumably to somehow prevent hybrid dysgenesis [78,79]. While harboring unpaired sister kinetochores could increase the chances of aneuploidy [45,46], it may allow metaphase I bivalents to switch between homologs being bi-oriented or sisters being bi-oriented in a non-hybrid or hybrid situation, respectively, to maintain optimal fertility in different scenarios.

Together, our data suggest that mechanisms of homolog recognition and pairing initiation are conserved across Lepidoptera and possibly more broadly across species, as transcription-related processes have been linked to pairing from yeast to mammals [6,49,51,53,54]. However, lineage-specific adaptations to the holocentric structure result in unique mechanisms of chromosome segregation between species, with moths limiting kinetochore formation to the center of chromosomes during spermatogenesis. This work highlights the importance of exploring fundamental processes in new model systems, as exploring new systems can lead to the discovery of new biology.

## Methods

### Insect strains and cell lines

Larvae of the *Savannah* and *Pi_bFog* strains were gifted from Erin Scully (USDA) and Arnaud Martin (GW), respectively, and stocks were then reared in the lab as previously described [37]. *Bombyx* larvae were obtained from FramsChams Panther Chameleons and reared as previously described [35]. PiD2 cells were a gift from Paul Shirk (USDA) and were grown in SF-900 ii serum-free cell culture media (Gibco) at 26°C.

### Genome and Oligopaint design

Oligopaints for ch5, 19, and 27 were designed using the scaffold-level *Plodia* genome assembly from the 2021 Ag100Pest consortium [38]. Since then, an updated chromosome-level assembly has been released as part of the same project (GCF_027563975.1) [39], which was used to design the Oligopaints for ch11 and 15. Oligopaint libraries were designed using a modified version of the Oligominer pipeline [80,81]. Oligos were designed to have 70–80 bp of homology and to label only unique, single copy sequences. Probe densities were adjusted to be approximately 1.5–2 oligos per kilobase (kb) of DNA. All information regarding genomic coordinates for Oligopaints can be found in S1 and S2 Tables. Oligo pools were purchased from Twist Biosciences (San Francisco, CA). Oligopaints were synthesized by adding index barcodes to each oligo for PCR-based amplification as previously described [82–84].

### Gene annotations and synteny mapping

We mapped the old Ag100Pest scaffolds to the new chromosome-level assembly using the *scaffold* tool of the *ragtag* software (v2.1.0) [85] with a minimum unique alignment length of 1000 bp and a minimum grouping confidence score of 0.5. Next, we validated that our chosen scaffolds are indeed complete chromosomes by generating a genome-wide alignment of both assemblies with the tool *nucmer* from the *mummer* software (v3.23) [86] followed by manual inspection of the pairwise alignments with the *mummer* tool *mummerplot*.

Gene predictions on the new *P*. *interpunctella* assembly were made by mapping existing gene annotations from the Ag100Pest assembly downloaded from GIGAdb [87] onto the new assembly [87], as described in https://github.com/TriLab-bioinf/LEI_ROSIN_2023. Briefly, mRNA sequences were generated from the fetched Ag100Pest *plo_final_annotation*.*gff* annotation file with the tool *gffread* (v0.12.7) [88] and then mapped onto the new assembly with *minimap2* (v2.26) [89] with the *-x splice* option. Mapping data was sorted by coordinate, converted to bam format with *samtools* (v.16.1) [90] and then transformed to gtf format with the scripts *sam_to_gtf*.*pl* and *get_genes_from_gtf*.*pl*. Next, same-stranded genes that overlapped at least 50% of their length were flagged as isoforms of the same gene with *merge_genes_from_gff*.*py* and *merge_overlapping_genes*.*pl* and redundant isoforms, sharing the same exonic structure, were removed with *remove_redundant_transcripts*.*pl*. Gene densities within a given probe domain were calculated by taking the total base pairs of DNA containing annotated genes within the probe domain divided by the probe size in base pairs (bp genes/bp probe).

Assessment of the degree of synteny between *B*. *mori* and the new *P*. *interpunctella* assemblies was performed as detailed in https://github.com/TriLab-bioinf/LEI_ROSIN_2023. Briefly, both genomic sequences were aligned to each other in protein space with the *mummer* tool *promer* and hit coordinates were then processed with the software *DAGchainer* [91] to identify syntenic blocks between the two genomes. Genome-wide synteny plots were generated in R (v4.1.1) with a customized version of *syntenyPlotteR* (v1.0.0) [92].

### Meiotic squashes, meiotic staging, IF, and DNA FISH on meiotic cells

For DNA FISH without IF, testes were harvested from 4^th^ - 5^th^ instar *Plodia* larvae (approximately 1 cm long) or 3^rd^ - 4^th^ instar *Bombyx* larvae (> 2 inches long), rinsed in 1X PBS, and incubated in 0.5% sodium citrate for 8 min. Testes were then transferred to siliconized coverslips and fixed with 45% acetic acid/1% PFA/1X PBS for 6 min. Using a poly-L-lysine coated glass slide, testes were then manually squashed, and slide/coverslip was flash frozen on dry ice. Coverslip was removed with a razor blade, and specimens were post-fixed in cold 3:1 methanol:glacial acetic acid for 10 min. Specimens were then washed thrice in 1X PBS-T (0.1% Triton-X 100; PBST^0.1^) and subjected to an ethanol row at -20°C (70%, 90%, 100% ethanol, 5 min each) before completely drying at room temperature (RT). Dried slides were left to cure at RT for approximately 3 d. After drying, slides were denatured at 72°C for 2.5 min in 2xSSCT/70% formamide before again being subjected to an ethanol row at -20°C. Slides were dried for 10 min at RT before applying primary Oligopaint probes in hybridization buffer cocktail. Hybridization buffer cocktail consisted of 25% hyb mix (10% dextran sulfate/2xSSCT/50% formamide/4% polyvinylsulfonic acid), 50% formamide, 20% polyvinylsulfonic acid, and RNaseA. After primary probes were sealed under a coverslip with rubber cement, slides were denatured at 92°C for 2.5 h and transferred to a humidified chamber at 37°C overnight. The next day, slides were washed in 2×SSCT at 60°C for 15 min, 2×SSCT at RT for 15 min, and 0.2×SSC at RT for 5 min. Fluorescently labeled secondary probes in hybridization buffer cocktail (no RNaseA) were then added to slides and incubated at 37°C for 2 h in a humidified chamber before repeating the above SSC washes. For images examining two chromosomes at a time by FISH (Fig 3C and 3D), distinct patterns of fluorescently labeled secondary oligos were used. Specifically, ch5 was labeled with the following secondaries: Arm1-alexa488, Center-atto565, Arm2-alexa647. Ch19 was labeled with: Arm1-alexa647, Arm2-atto565. Slides were stained with DAPI and mounted in Prolong Diamond (Invitrogen/ThermoFisher, Waltham, MA). During imaging, meiotic stages were determined based on DAPI morphology and chromosome/Oligopaint morphology as previously described [35].

For IF on testes squashes, testes were dissected at RT in SF-900 media, rinsed in PBS, and fixed in 4% PFA for 10 min on a siliconized coverslip. After fixation, testes were manually squashed between the siliconized coverslip and a poly-L-lysine coated glass slide. Slides were then snap frozen before removing coverslips and washing in PBS then PBST^0.1^ for 5 min each. Slides were permeabilized in PBST^0.5^ for 15 min at RT before blocking in 2% BSA in PBST^0.1^ for 1 h at RT. Primary antibodies were diluted in blocking solution to the below concentrations and incubated on slides under parafilm coverslips overnight at 4°C. The next day, slides were washed thrice in PBS-T 0.1% before adding secondary antibodies for a 1 h RT incubation. After washing off secondary antibodies, slides were DAPI stained, washed thrice in PBST^0.1^, mounted in Prolong Diamond, and left to cure at RT for at least 24 h. Slides were then sealed with nail polish before imaging. For IF with tubulin or SMC1 antibodies, cells had an additional permeabilization in 100% methanol at RT for 20 minutes after PBST^0.5^ before proceeding with blocking. For SMC1 only, blocking solution also contained 0.1% digitonin in addition to 0.1% Triton-X 100.

For IF/FISH, after washing off secondary antibodies, slides were post-fixed in 4% PFA for 10 min before being subjected to the following pre-denaturation procedure: 2X SSCT 10 min at RT, 20% formamide in 2X SSCT 10 min at RT, 50% formamide in 2X SSCT (50% FM) 10 min at RT, 50% FM at 37°C 2 hours, 50% FM at 92°C 3 min, 50% FM at 60°C 20 min. After pre-denaturation, primary Oligopaint probes were applied in hybridization buffer cocktail as described above and sealed under a glass coverslip using rubber cement. Slides were denatured 2.5 min at 92°C and incubated at 37°C overnight. The next day, secondary probes were applied exactly as described above before DAPI staining and mounting in Prolong Diamond.

The following antibodies and dilutions were used for IF: rabbit anti-Dsn1 1/1000, rabbit anti-CENPT 1/1000, and rabbit anti-Spc24/25 1/1000 (gift from Ines Drinnenberg [47]), rat anti-SMC1 1/250 (gift from Scott Hawley [93]), anti-tubulin cocktail (Synaptic Systems 302 209 1/500 + sigma T6074-200 1/500). The following secondary antibodies were all from Invitrogen and used at 1/500: donkey anti-mouse 488 (A21202), donkey anti-mouse 555 (A31570), donkey anti-mouse 647 (A31571), donkey anti-rabbit 488 (A21206), donkey anti-rabbit 555 (A31572), donkey anti-rabbit 647 (A31573), donkey anti-chicken 488 (A78948), and donkey anti-rat 647 plus (A48272).

### PiD2 mitotic spreads and IF

For mitotic arrest, colcemid (ThermoFisher) was added directly to a flask of log-phase cells at a concentration of 0.1 ug/ml. Cells were incubated 2–3 h at 26°C. 2.5x10^5^ cells were harvested and resuspended in 0.5% sodium citate for 10 min. Spreads were then made using a Shandon Cytospin 4 (ThermoFisher) at 600 or 1200 rpm for 5 min with high acceleration. Slides were subsequently fixed in 4% PFA for 10 min, permeabilized in PBST^0.5^ for 15 min, and blocked in 2% BSA at RT for 1 h. IF on spreads was then performed as previously described [94, 95]. Briefly, primary antibodies were incubated at 4°C overnight (rabbit anti-Dsn1 1/1000). Slides were washed thrice in PBST^0.1^ before adding secondary antibodies (donkey anti-rabbit 488 1/500 (Invitrogen A21206)), which were incubated at RT for 1 h. After washing in PBST^0.1^, slides were stained with DAPI and mounted in Prolong Diamond.

### Imaging, quantification, and data analysis

Ch5, 19, and 27 Oligopaints on testes squashes were imaged on a Leica DMi6000 wide-field inverted fluorescence microscope using an APO 63x/1.40 Oil objective (Leica Biosystems, Buffalo Grove, IL), Leica DFC9000 sCMOS Monochrome Camera, EL6000 light source, and LasX software. Ch11 and 15 Oligopaints on testes squashes and PiD2 mitotic spreads were imaged on a Leica DMi8 wide-field inverted fluorescence microscope using an APO 63x/1.40 CS2 oil objective (Leica Biosystems, Buffalo Grove, IL), K8 CMOS Monochrome Camera, LED8 light source, and LasX software. Widefield images were post-processed using Huygens deconvolution software (SVI, Hilversum, Netherlands) or Leica Thunder deconvolution. IF and IF/FISH were imaged on a Stellaris 8 laser-scanning confocal with HyD S detectors and Lightning super-resolution processing (Leica Biosystems, Buffalo Grove, IL). All quantification was performed manually. Statistical analyses were performed in Prism 10. Tiffs and line scans were created in ImageJ.

## Supporting information

S1 Fig Genome alignments between *Plodia* genome versions or *Plodia* and *Bombyx mori*. A) Alignment between 2021 *Plodia* genome (bottom) used to design Oligopaints for ch5, 19, and 17, and new 2023 release of *Plodia* genome (top) used for designing Oligopaints for ch11 and ch15. B-F) Chromosome-specific alignment between 2021 *Plodia* genome (bottom) used to design Oligopaints and new 2023 release of *Plodia* genome (top) for the five chromosomes analyzed in this manuscript. Different colors indicate different syntenic blocks. Chromosome or scaffold coordinates are indicated above and below. G) Alignment between latest release of the *Bombyx mori* genome (top) and 2023 *Plodia* genome release (bottom).
(TIF)

S2 Fig Comparison of *Bombyx* and *Plodia* pairing initiation and metaphase chromosome configurations. A) Scatter plot showing Arm1:Arm2 pairing initiation ratio (X-axis) versus Arm1:Arm2 gene density ratio (Y-axis) for *Bombyx* chromosomes (gray) and *Plodia* chromosomes (blue). *Bombyx* “Arm1” and “Arm2” were previously referred to as “tel1” and “tel2”, respectively [35]. Linear regression used to calculate the line of best fit, R^2^, and *p*-value. B) Bar graph showing quantification of metaphase I orientation for *Plodia* ch5, 11, 15, 19, and 27 as shown in Fig 3B compared to *Bombyx* data for chromosomes 7, 15, 16, and 23 from [35]. *Bombyx* ch7 and 15 are orthologous to *Plodia* ch19 and 5, respectively.
(TIF)

S3 Fig Cells at diakinesis show chromosome structures consistent with both telomere-oriented and center-oriented bivalents. A) Representative prophase I cells at Diakinesis substage labeled with ch5 Oligopaints. Arm1 is shown in cyan and Arm2 is shown in magenta. Cells 1 and 2 show structures consistent with telomere-oriented metaphase I bivalents. Cells 3 and 4 show structures consistent with center-oriented metaphase I bivalents. DAPI is shown in gray. Scale bar equals 5 μm. B) Representative prophase I cell at Diakinesis substage labeled with a FISH probe recognizing the insect pentameric telomere repeat (shown in green). DAPI is shown in gray. Scale bar equals 5 μm.
(TIF)

S4 Fig *Plodia* ch11 has center-localized kinetochores during meiosis, but all chromosomes are holocentric during mitosis. A) Representative pachytene cell labeled with ch5 Oligopaints where both Arm probes are the same color (magenta) and the Center is yellow, as in Fig 4. Schematic of paints is shown above. Scale bar is 5 μm. Dashed line approximates the nuclear edge. B) Widefield image of center-oriented chromosome from *Plodia* 5^th^ instar larval testis squash labeled with ch5 Oligopaints shown in A, and Dsn1 IF (green). DAPI is shown in blue. Scale bar is 5 μm. Cartoon schematic of painted bivalent is shown to the right. Asterisks indicate the location of spindle poles at metaphase I. C) Representative metaphase I cell from *Plodia* 5^th^ instar larval testes squashes labeled with ch11 Oligopaints and Dsn1 IF showing a telomere-oriented chromosome. Schematic for Oligopaints is shown above. Arm1 and Arm2 probes are shown in magenta, Center probe in yellow, Dsn1 IF is shown in green, and DAPI in blue. Scale bar = 5 μm. Cartoon schematic of bivalents are shown on the right. Asterisks indicate the spindle poles. Asterisks indicate the location of spindle poles at metaphase I. D) Mitotic metaphase chromosome spreads from PiD2 cultured cells labeled with DAPI (blue) and Dsn1 (green). Scale bar equals 5 μm. Inset zooms are shown to the right.
(TIF)

S5 Fig *Bombyx* chromosomes are also center-kinetic during spermatogenesis. Top: Schematic of *Bombyx* ch15 Oligopaints used in this experiment. Bottom: Representative metaphase I cells from *Bombyx* 5^th^ instar larval testes squashes labeled with ch15 Oligopaints and Dsn1 IF showing telomere-oriented chromosomes. Arm1 and Arm2 probes are shown in magenta, Center probe in yellow, Dsn1 IF is shown in green, and DAPI in blue. Scale bar = 2.5 μm. Columns 2–5 are zooms of painted chromosome in column 1, rotated 90 degrees. Asterisks indicate the direction of the spindle poles.
(TIF)

S6 Fig CENP-T localizes along the chromosome axis at pachytene and is restricted to central regions by metaphase I. A) Representative IF images showing the localization of CENP-T kinetochore protein throughout prophase I of *Plodia* spermatogenesis. Stages shown are indicated on the left. DAPI is shown in magenta. CENP-T is shown in cyan. B) Representative IF showing co-localization of CENP-T with the SMC1 subunit of cohesin along the chromosome axis at pachytene in *Plodia*. SMC1 is shown in yellow. C) Representative metaphase I cell from *Plodia* larval testis squashes showing an approximately central localization of CENP-T kinetochore protein (away from chromosome ends). DAPI is shown in magenta. CENP-T is shown in cyan. D) Representative IF images showing the localization of Dsn1 kinetochore protein throughout early prophase I from *Plodia* larval testis squashes. Stages shown are indicated on the left. DAPI is shown in magenta. Dsn1 is shown in green. E) Representative *Bombyx* metaphase I cell from larval testis squash showing an approximately central localization of Spc24/25 kinetochore protein (part of the Ncd80 complex) away from chromosome ends. DAPI is shown in magenta. Spc24/25 is shown in white. Arrows in left panel indicate zoomed chromosomes shown below. Scale bar = 5 μm in A-E.
(TIF)

S7 Fig *Bombyx* chromosomes still show telomere-oriented configurations at early anaphase I. A-B) Representative early (**A**) and late (**B**) anaphase I cells from *Bombyx* 5^th^ instar larval testes squashes labeled with ch15 Oligopaints and Dsn1 IF. Schematic for Oligopaints is shown above in **A**. Arm1 and Arm2 probes are shown in magenta, Center probe in yellow, Dsn1 IF is shown in green, and DAPI in blue. Scale bar = 2.5 μm. Asterisks indicate the direction of the spindle poles. Dashed line indicates approximate cell border. C) Model for chromosome orientation flipping in late anaphase. Arm1 and Arm2 probes are shown in magenta, Center probe in yellow, Dsn1 IF is shown in green, and spindle microtubules are shown in blue.
(TIF)

S1 Table Probe coordinates for ch5, 19, and 27 based on 2021 *Plodia* genome assembly. Genome coordinates for paints for Plodia ch5, 19, and 27, as well as the paint size (amount of genome being painted), probe density of oligos, and size of oligos.
(DOCX)

S2 Table Probe coordinates for ch11 and 15 based on 2022 *Plodia* genome assembly. Genome coordinates for paints for Plodia ch11 and 15, as well as the paint size (amount of genome being painted), probe density of oligos, and size of oligos.
(DOCX)

S1 Data Final data file. Data used to generate graphs in all main and supplementary images.
(XLSX)
