# Supplementary figures and images for: Chromosome segregation during spermatogenesis occurs through a unique center-kinetic mechanism in holocentric moth species
Source: PLoS Genet. 2024 Jun 24;20(6):e1011329. doi: 10.1371/journal.pgen.1011329 (PMC11226059; doi:10.1371/journal.pgen.1011329)

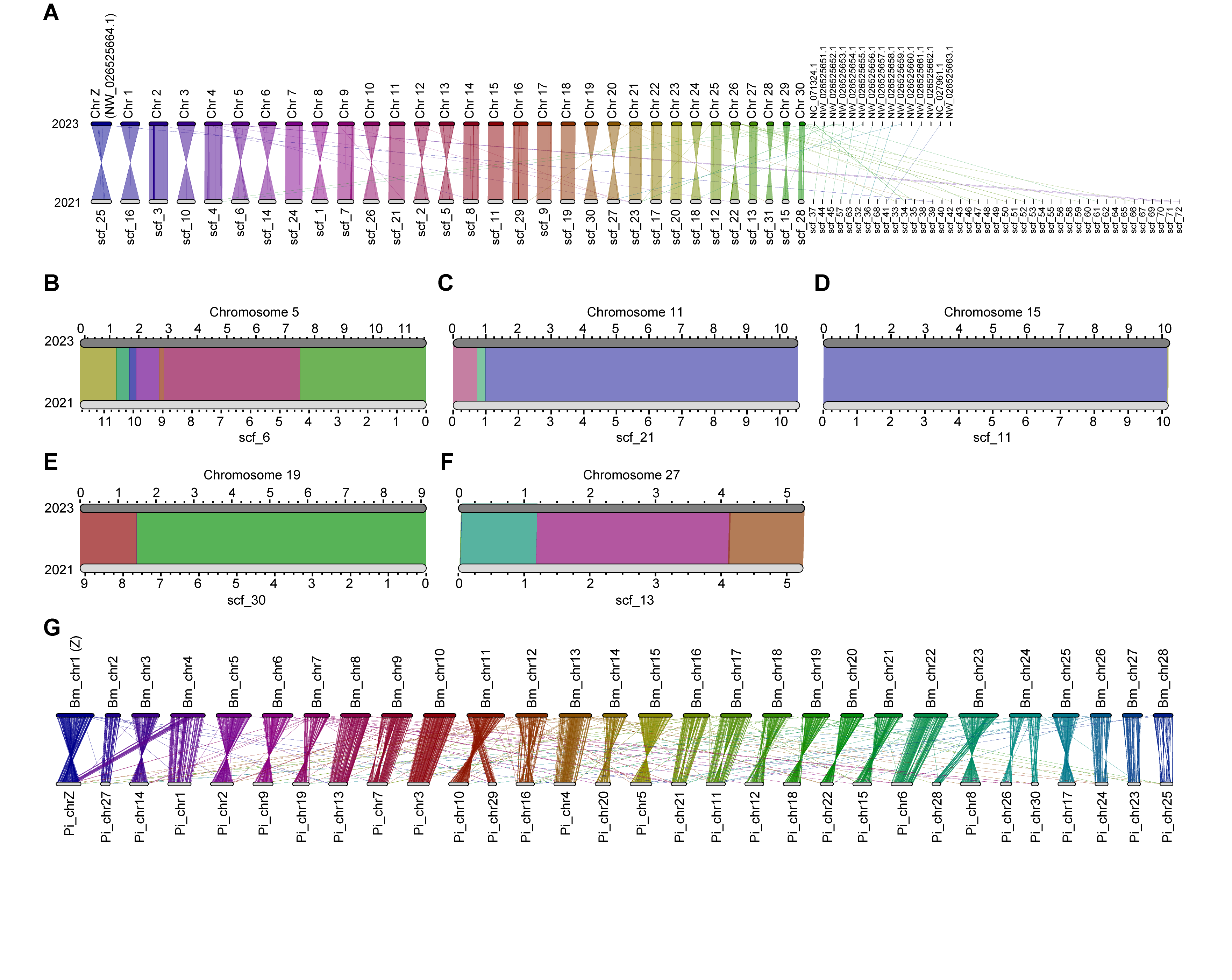

Supplement: S1 Fig — A) Alignment between 2021 Plodia genome (bottom) used to design Oligopaints for ch5, 19, and 17, and new 2023 release of Plodia genome (top) used for designing Oligopaints for ch11 and ch15. B-F) Chromosome-specific alignment between 2021 Plodia genome (bottom) used to design Oligopaints and new 2023 release of Plodia genome (top) for the five chromosomes analyzed in this manuscript. Different colors indicate different syntenic blocks. Chromosome or scaffold coordinates are indicated above and below. G) Alignment between latest release of the Bombyx mori genome (top) and 2023 Plodia genome release (bottom). (TIF) [file pgen.1011329.s001.tif]

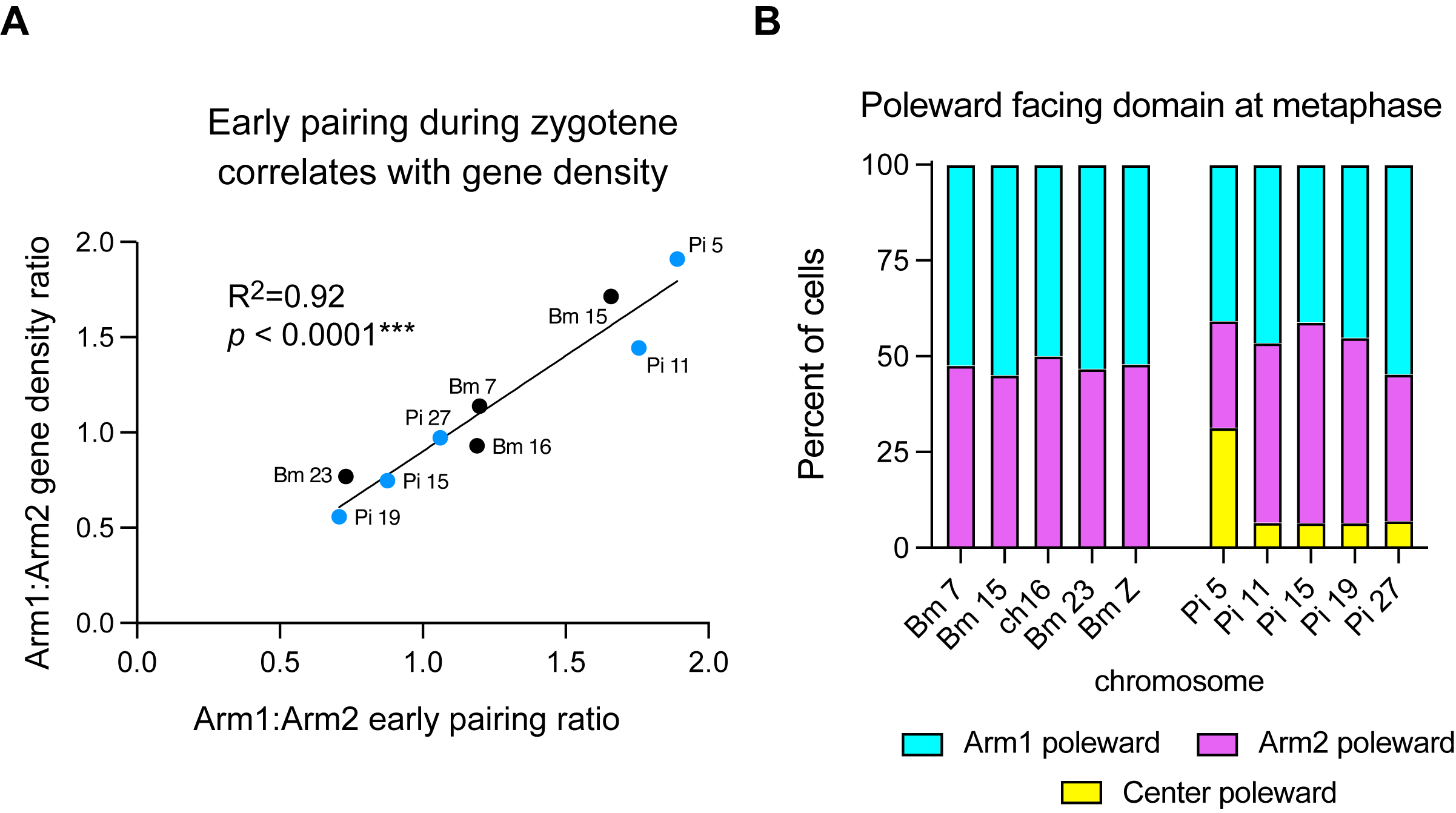

Supplement: S2 Fig — A) Scatter plot showing Arm1:Arm2 pairing initiation ratio (X-axis) versus Arm1:Arm2 gene density ratio (Y-axis) for Bombyx chromosomes (gray) and Plodia chromosomes (blue). Bombyx “Arm1” and “Arm2” were previously referred to as “tel1” and “tel2”, respectively [35]. Linear regression used to calculate the line of best fit, R2, and p-value. B) Bar graph showing quantification of metaphase I orientation for Plodia ch5, 11, 15, 19, and 27 as shown in Fig 3B compared to Bombyx data for chromosomes 7, 15, 16, and 23 from [35]. Bombyx ch7 and 15 are orthologous to Plodia ch19 and 5, respectively. (TIF) [file pgen.1011329.s002.tif]

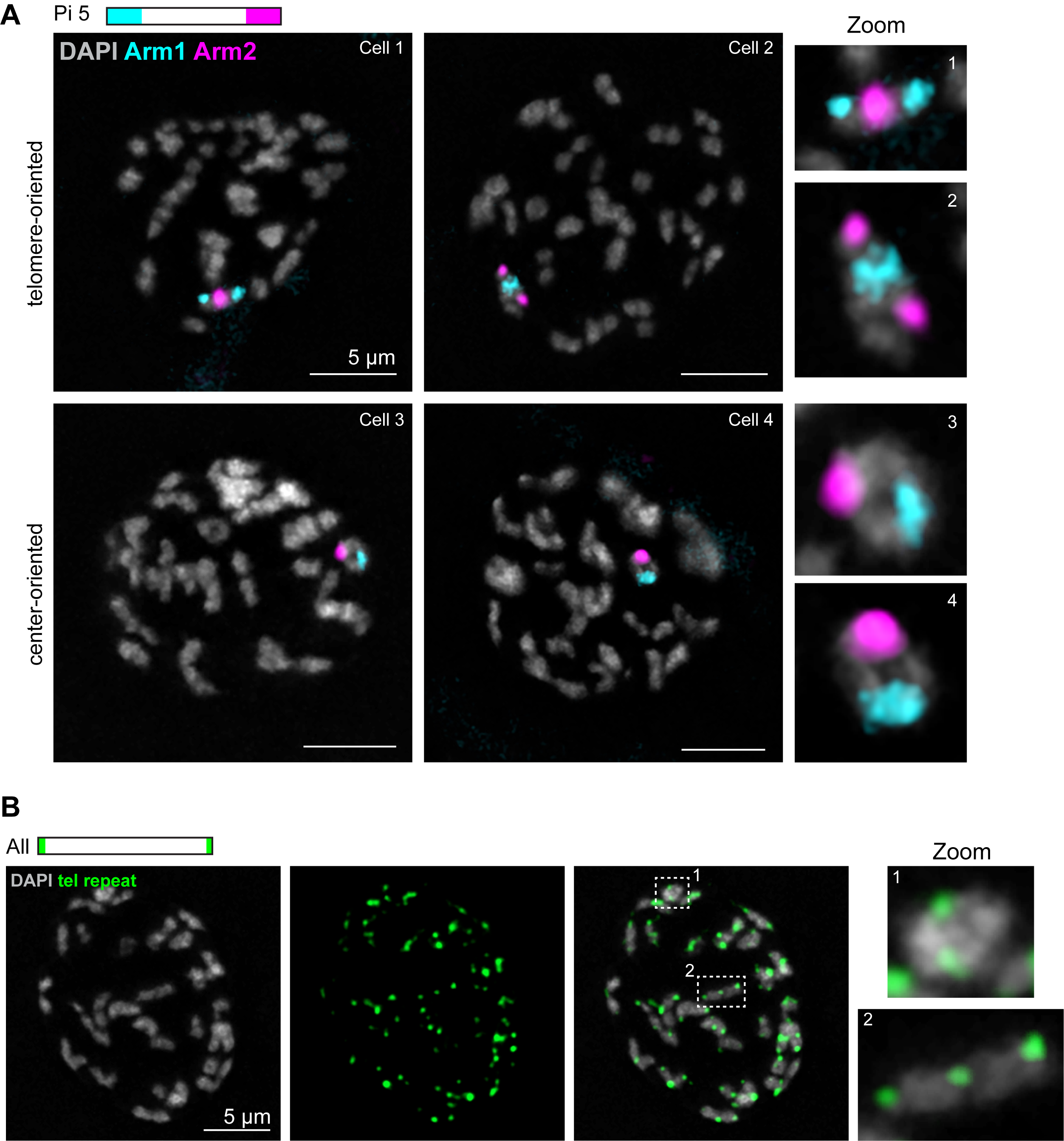

Supplement: S3 Fig — A) Representative prophase I cells at Diakinesis substage labeled with ch5 Oligopaints. Arm1 is shown in cyan and Arm2 is shown in magenta. Cells 1 and 2 show structures consistent with telomere-oriented metaphase I bivalents. Cells 3 and 4 show structures consistent with center-oriented metaphase I bivalents. DAPI is shown in gray. Scale bar equals 5 μm. B) Representative prophase I cell at Diakinesis substage labeled with a FISH probe recognizing the insect pentameric telomere repeat (shown in green). DAPI is shown in gray. Scale bar equals 5 μm. (TIF) [file pgen.1011329.s003.tif]

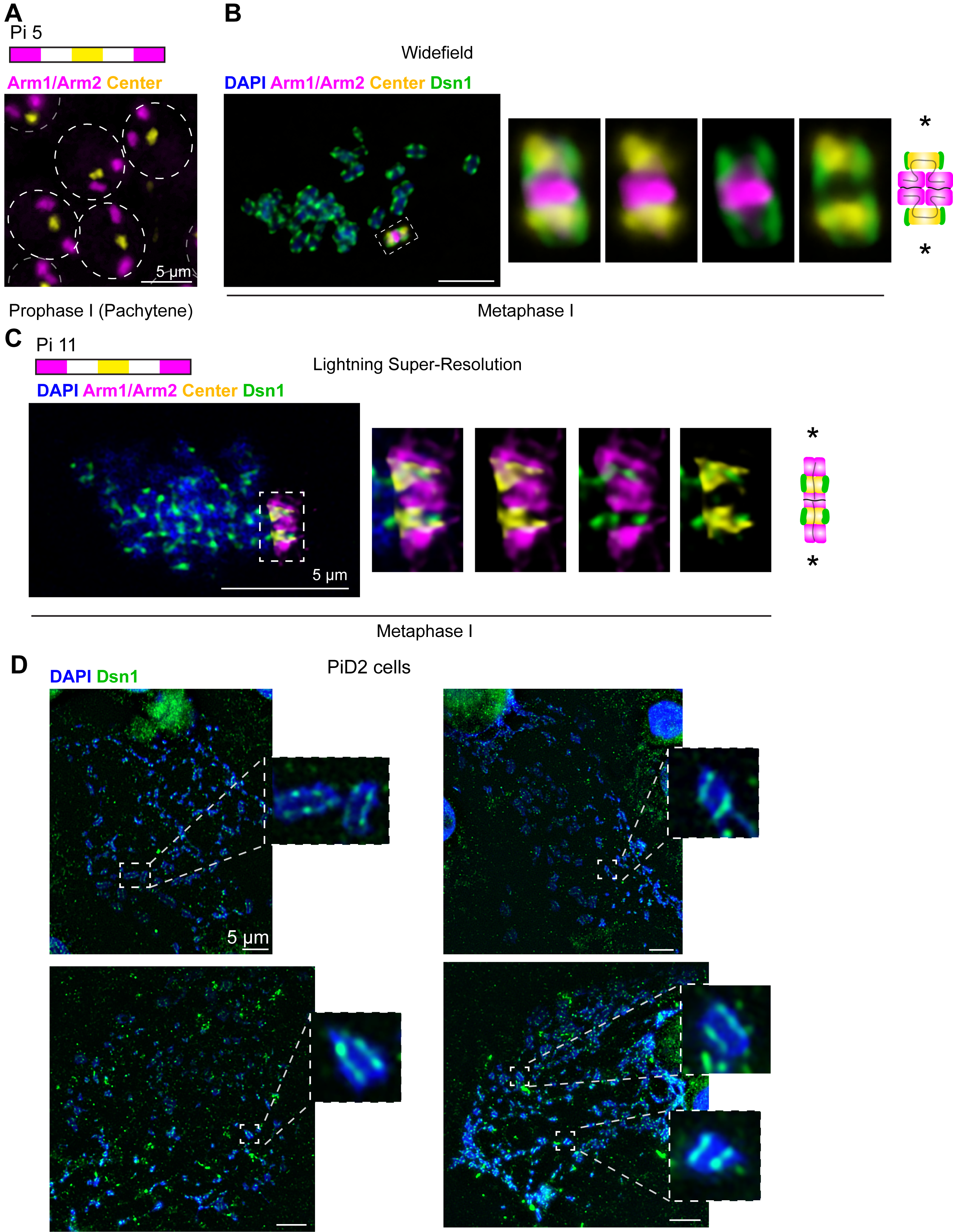

Supplement: S4 Fig — A) Representative pachytene cell labeled with ch5 Oligopaints where both Arm probes are the same color (magenta) and the Center is yellow, as in Fig 4. Schematic of paints is shown above. Scale bar is 5 μm. Dashed line approximates the nuclear edge. B) Widefield image of center-oriented chromosome from Plodia 5th instar larval testis squash labeled with ch5 Oligopaints shown in A, and Dsn1 IF (green). DAPI is shown in blue. Scale bar is 5 μm. Cartoon schematic of painted bivalent is shown to the right. Asterisks indicate the location of spindle poles at metaphase I. C) Representative metaphase I cell from Plodia 5th instar larval testes squashes labeled with ch11 Oligopaints and Dsn1 IF showing a telomere-oriented chromosome. Schematic for Oligopaints is shown above. Arm1 and Arm2 probes are shown in magenta, Center probe in yellow, Dsn1 IF is shown in green, and DAPI in blue. Scale bar = 5 μm. Cartoon schematic of bivalents are shown on the right. Asterisks indicate the spindle poles. Asterisks indicate the location of spindle poles at metaphase I. D) Mitotic metaphase chromosome spreads from PiD2 cultured cells labeled with DAPI (blue) and Dsn1 (green). Scale bar equals 5 μm. Inset zooms are shown to the right. (TIF) [file pgen.1011329.s004.tif]

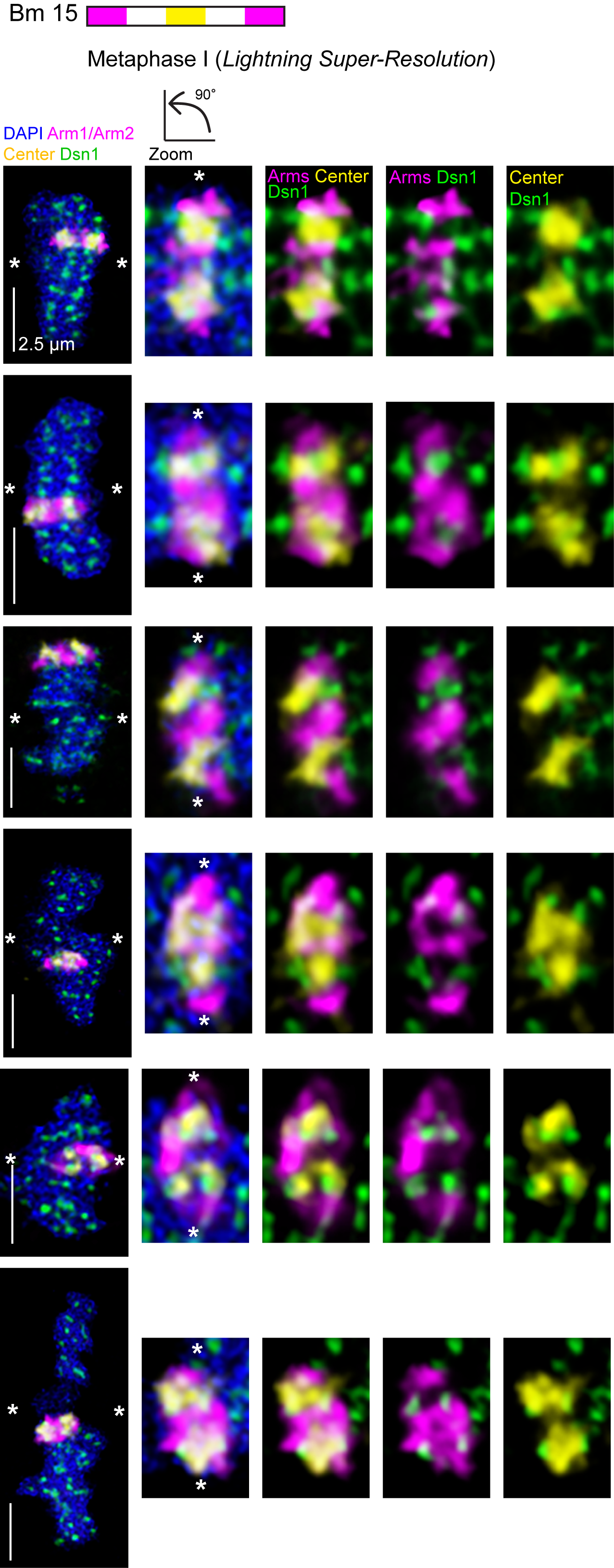

Supplement: S5 Fig — Top: Schematic of Bombyx ch15 Oligopaints used in this experiment. Bottom: Representative metaphase I cells from Bombyx 5th instar larval testes squashes labeled with ch15 Oligopaints and Dsn1 IF showing telomere-oriented chromosomes. Arm1 and Arm2 probes are shown in magenta, Center probe in yellow, Dsn1 IF is shown in green, and DAPI in blue. Scale bar = 2.5 μm. Columns 2–5 are zooms of painted chromosome in column 1, rotated 90 degrees. Asterisks indicate the direction of the spindle poles. (TIF) [file pgen.1011329.s005.tif]

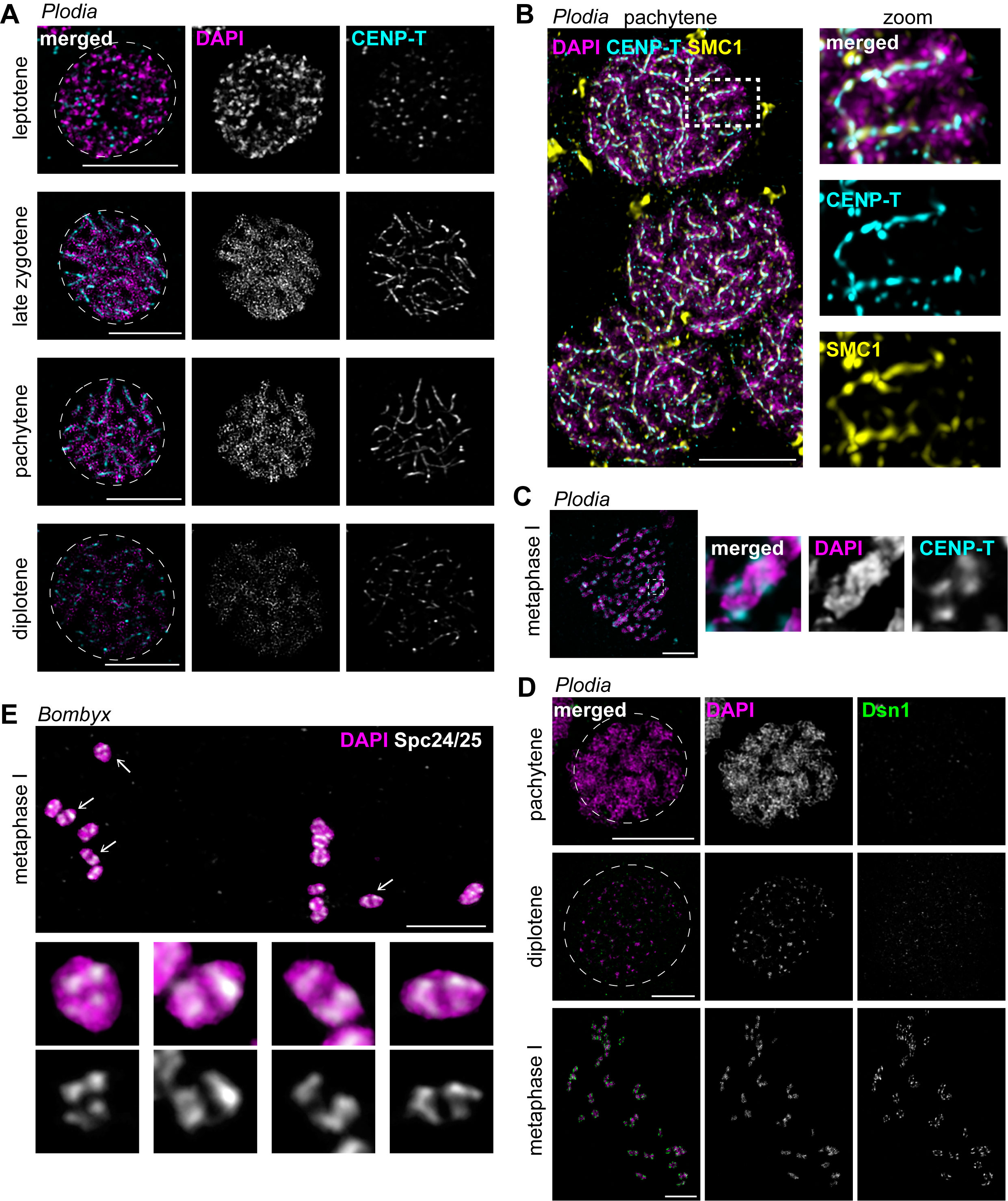

Supplement: S6 Fig — A) Representative IF images showing the localization of CENP-T kinetochore protein throughout prophase I of Plodia spermatogenesis. Stages shown are indicated on the left. DAPI is shown in magenta. CENP-T is shown in cyan. B) Representative IF showing co-localization of CENP-T with the SMC1 subunit of cohesin along the chromosome axis at pachytene in Plodia. SMC1 is shown in yellow. C) Representative metaphase I cell from Plodia larval testis squashes showing an approximately central localization of CENP-T kinetochore protein (away from chromosome ends). DAPI is shown in magenta. CENP-T is shown in cyan. D) Representative IF images showing the localization of Dsn1 kinetochore protein throughout early prophase I from Plodia larval testis squashes. Stages shown are indicated on the left. DAPI is shown in magenta. Dsn1 is shown in green. E) Representative Bombyx metaphase I cell from larval testis squash showing an approximately central localization of Spc24/25 kinetochore protein (part of the Ncd80 complex) away from chromosome ends. DAPI is shown in magenta. Spc24/25 is shown in white. Arrows in left panel indicate zoomed chromosomes shown below. Scale bar = 5 μm in A-E. (TIF) [file pgen.1011329.s006.tif]

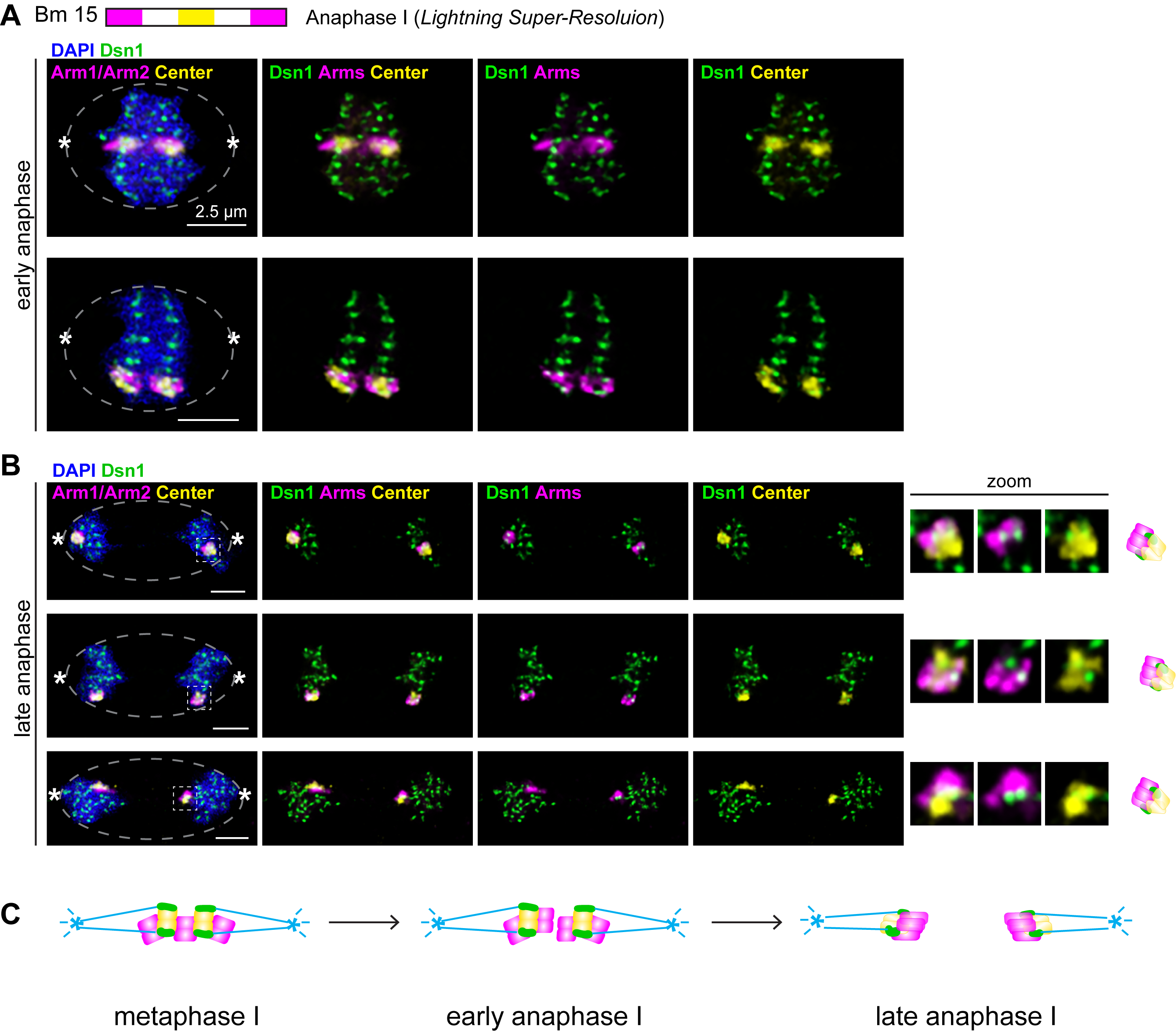

Supplement: S7 Fig — A-B) Representative early (A) and late (B) anaphase I cells from Bombyx 5th instar larval testes squashes labeled with ch15 Oligopaints and Dsn1 IF. Schematic for Oligopaints is shown above in A. Arm1 and Arm2 probes are shown in magenta, Center probe in yellow, Dsn1 IF is shown in green, and DAPI in blue. Scale bar = 2.5 μm. Asterisks indicate the direction of the spindle poles. Dashed line indicates approximate cell border. C) Model for chromosome orientation flipping in late anaphase. Arm1 and Arm2 probes are shown in magenta, Center probe in yellow, Dsn1 IF is shown in green, and spindle microtubules are shown in blue. (TIF) [file pgen.1011329.s007.tif]
