# Supplementary material for: Chromosome segregation during spermatogenesis occurs through a unique center-kinetic mechanism in holocentric moth species
Source: PLoS Genet. 2024 Jun 24;20(6):e1011329. doi: 10.1371/journal.pgen.1011329 (PMC11226059; doi:10.1371/journal.pgen.1011329)
Supplement: S1 Table — Genome coordinates for paints for Plodia ch5, 19, and 27, as well as the paint size (amount of genome being painted), probe density of oligos, and size of oligos. (DOCX) [file pgen.1011329.s008.docx]

**Table S1. Probe coordinates for ch5, 19, and 27 based on 2021 *Plodia* genome assembly**

| **probe** | **start** | **stop** | **size (Mbp)** | **Average probe density (probes/kb)** | **Oligo size (nt)** |
| --- | --- | --- | --- | --- | --- |
| ch5 arm1 | 17108 | 2372801 | 2.36 | 1.5 | 70 |
| ch5 center | 4728496 | 7084189 | 2.36 | 1.5 | 70 |
| ch5 arm2 | 9439884 | 11795576 | 2.36 | 1.5 | 70 |
| ch19 arm1 | 5270 | 1828708 | 1.82 | 1.5 | 70 |
| ch19 center | 3652148 | 5475586 | 1.82 | 1.5 | 70 |
| ch19 arm2 | 7299026 | 9122466 | 1.82 | 1.5 | 70 |
| ch27 arm1 | 50288 | 1083390 | 1.03 | 1.5 | 70 |
| ch27 center | 2116492 | 3149595 | 1.03 | 1.5 | 70 |
| ch27 arm2 | 4182697 | 5215799 | 1.03 | 1.5 | 70 |
