# Supplementary material for: Chromosome segregation during spermatogenesis occurs through a unique center-kinetic mechanism in holocentric moth species
Source: PLoS Genet. 2024 Jun 24;20(6):e1011329. doi: 10.1371/journal.pgen.1011329 (PMC11226059; doi:10.1371/journal.pgen.1011329)
Supplement: S2 Table — Genome coordinates for paints for Plodia ch11 and 15, as well as the paint size (amount of genome being painted), probe density of oligos, and size of oligos. (DOCX) [file pgen.1011329.s009.docx]

**Table S2. Probe coordinates for ch11 and 15 based on 2022 *Plodia* genome assembly**

| **Probe** | **Start** | **Stop** | **Size (Mbp)** | **Average Probe Density (probes/kb)** | **Oligo size (nt)** |
| --- | --- | --- | --- | --- | --- |
| ch11 arm1 | 19950 | 2111443 | 2.09 | 2 | 80 |
| ch11 center | 4222887 | 6334330 | 2.11 | 2 | 80 |
| ch11 arm2 | 8445774 | 10549111 | 2.10 | 2 | 80 |
| ch15 arm1 | 75295 | 2045295 | 1.97 | 2 | 80 |
| ch15 center | 4090592 | 6135886 | 2.05 | 2 | 80 |
| ch15 arm2 | 8181183 | 10154850 | 1.97 | 2 | 80 |
